# Time lags in the regulation of symbiotic nitrogen fixation

**DOI:** 10.1111/nph.70295

**Published:** 2025-06-11

**Authors:** Thomas A. Bytnerowicz, Kevin L. Griffin, Duncan N. L. Menge

**Affiliations:** ^1^ Department of Ecology, Evolution and Environmental Biology Columbia University New York NY 10027 USA; ^2^ Department of Integrative Biology University of Texas at Austin Austin TX 78712 USA; ^3^ Department of Earth and Environmental Sciences Columbia University Palisades NY 10964 USA

**Keywords:** actinorhizal, facultative, legume, nitrogen limitation, rhizobial, symbiosis, temperature

## Abstract

- Theory has shown that time lags in the regulation of symbiotic nitrogen (N) fixation (SNF) can be important to the competitive dynamics and ecosystem consequences of N‐fixing trees, but measurements of these time lags are lacking.
- Here, we used a novel method to measure SNF in seedlings of four N‐fixing tree species that represent tropical and temperate origins and actinorhizal and rhizobial symbiotic associations, each grown under warm and cold temperature regimes. We added N to previously N‐poor pots to induce downregulation and flushed N out of previously N‐rich pots to induce upregulation.
- It took 31–51 d for SNF to decline by 95%, with faster downregulation in temperate species and at warm temperatures. Upregulation by 95% took 108–138 d in total, including 21–57 d after SNF was first detectable. SNF started earlier in rhizobial symbioses, but increased faster once it started in actinorhizal symbioses.
- These results suggest that time lags in regulating SNF represent a significant constraint on facultative SNF and can lead to large losses of available N from ecosystems, providing a resolution to the paradox of sustained N richness.

## Introduction

Symbiotic nitrogen (N)‐fixing plants are the dominant natural source of new N in many ecosystems (Cleveland *et al*., 2013; Staccone *et al*., 2020), capable of supplying more than 100 kg N ha^−1^ yr^−1^ (Binkley *et al*., 1994) and are co‐dominant with other sources globally (Davies‐Barnard & Friedlingstein, 2020; Reis Ely *et al*., 2025). Symbiotic N fixation (SNF) can play a key role in forest succession (Chapin *et al*., 1994; Batterman *et al*., 2013a; Sullivan *et al*., 2014; Levy‐Varon *et al*., 2019), in the ability of forests to sequester carbon as atmospheric CO_2_ levels rise (Hungate *et al*., 2003; Goll *et al*., 2012; Wieder *et al*., 2015; Sulman *et al*., 2019), and in losses of reactive N via gaseous emissions (Erickson & Perakis, 2014; Kou‐Giesbrecht & Menge, 2021) and leaching (Binkley *et al*., 1992; Compton *et al*., 2003).

There is evidence that many N‐fixing trees are capable of regulating SNF via a facultative SNF strategy (Barron *et al*., 2011; Batterman *et al*., 2013b; Bauters *et al*., 2016; Dovrat *et al*., 2018, 2020; Taylor & Menge, 2018; McCulloch & Porder, 2021): increasing or decreasing SNF in response to changes in N supply relative to N demand (n.b. Menge *et al*., 2023). According to theory, one of the pivotal aspects of facultative SNF is the speed at which N fixers adjust SNF (Menge *et al*., 2009). Adjustments that are slow relative to changes in N supply or N demand create periods of under‐fixation (less SNF than is necessary to alleviate N limitation to plant growth) and over‐fixation (vice versa). Both under‐fixation and over‐fixation may reduce the growth rate and competitive ability of a facultative N fixer relative to its neighbors, independent of a cost of being facultative (Menge *et al*., 2009). This reduction in growth and competitive ability results from N limitation when plants are under‐fixing and the carbon cost of SNF (Tjepkema & Winship, 1980; Gutschick, 1981) when plants are over‐fixing.

The insight that time lags limit the success of phenotypic plasticity (facultative SNF is an example of phenotypic plasticity) is well known in evolutionary ecology (Padilla & Adolph, 1996; DeWitt *et al*., 1998; Auld *et al*., 2010), which might have consequences for other aspects of SNF strategies. For example, Menge *et al*. (2015) demonstrated that some Mediterranean legumes downregulated SNF before their own N demand was fully met (i.e. over‐regulation of SNF) or incompletely downregulated SNF when N was no longer limiting, which may be evolved responses to time lags. By over‐regulating SNF, lagged N‐fixing plants can avoid the perils of over‐fixation. Conversely, by incompletely downregulating, N‐fixing plants can more rapidly increase SNF if N becomes limiting again or fix excess N to build up N reserves (analogous to luxury consumption via N uptake from the soil; Chapin, 1980). The fact that incomplete downregulation is often observed (Taylor & Menge, 2018; Menge *et al*., 2023) or inferred (Schmidt *et al*., 2023) where light availability is high supports this idea: It is easier to hedge bets and sustain low levels of N fixation when there is ample energy available to pay the costs of SNF.

At the ecosystem scale, time lags in facultative SNF have been hypothesized as a mechanism causing the paradox of N richness in tropical forests (Hedin *et al*., 2009), which are often characterized by high sustained export of available N (Brookshire *et al*., 2012) despite likely downregulation of SNF within individual trees (Barron *et al*., 2011; Batterman *et al*., 2013a). Although other hypotheses have also been proposed to explain the tropical forest N paradox, including horizontal heterogeneity in SNF (Menge & Levin, 2017), vertical heterogeneity in N availability that creates conditions favorable for N fixation by epiphytic or free‐living N fixers in leaf litter or tree canopies (Reed *et al*., 2008; Hedin *et al*., 2009; Menge & Hedin, 2009; Cleveland *et al*., 2022), and incomplete downregulation of SNF (Menge *et al*., 2015), these are not mutually exclusive, and the time lag hypothesis is relatively untested. Theory says that time lags in SNF regulation longer than 1 d to 1 wk can lead to high exports of plant‐available N (tens of kg N ha^−1^ yr^−1^) from ecosystems (Menge *et al*., 2009).

Time lags may also contribute to the latitudinal gradient in N‐fixing tree abundance (Menge *et al*., 2014). N fixers should have a competitive advantage where N is limiting, because they can access external N, but they should have a disadvantage where N is not limiting, because SNF carries a carbon cost (Gutschick, 1981; Vitousek & Howarth, 1991). Higher latitude forests are generally more N limited than tropical forests (Vitousek & Sanford, 1986; McGroddy *et al*., 2004; Hedin *et al*., 2009; Brookshire *et al*., 2012; Du *et al*., 2020; but see Wright, 2019), suggesting that N fixers should have an advantage at higher latitudes. However, at least among trees, the data show the opposite trend: N‐fixing tree abundance frequently reaches 10–20% in tropical forests, but N‐fixing trees are effectively absent at higher latitudes (ter Steege *et al*., 2006; Menge *et al*., 2014, 2017, 2019; Gei *et al*., 2018; Steidinger *et al*., 2019). If time lags are sufficiently longer at lower temperatures or in the N‐fixing tree type that is more common at higher latitudes (actinorhizal, as opposed to rhizobial; Menge *et al*., 2014, 2017), facultative strategies may be outcompeted by obligate strategies at high latitudes. Menge *et al*. (2014) showed that N‐fixing trees that do not regulate SNF (obligate N fixers) in response to changing N supply or demand can result in lower N‐fixing tree abundance. This results from obligate SNF increasing ecosystem N supply to a level at which N fixers are competitively excluded, in contrast to facultative N fixers, which can stabilize N supply and maintain dominance or coexist with nonfixers (Bytnerowicz & Menge, 2021).

Although Chen & Phillips (1977) demonstrated that SNF is downregulated in *Pisum sativum* (garden peas) within 1 wk upon application of ammonium and nitrate, the time it takes for SNF to be regulated in wild N‐fixing plants, including tree species, is unknown. Furthermore, it is unclear how quickly SNF can be upregulated following reductions in N availability in any plant–bacterial symbioses. We can, however, speculate about the relative magnitude of SNF time lags. First, downregulation of SNF is likely to be faster than upregulation because plant regulation of O_2_ supply to a nodule, one mechanism by which nodules are sanctioned in rhizobial symbioses (Kiers *et al*., 2003), is likely to be faster than the time for constructing new functional nodules. Second, time lags may vary by symbiotic association, as actinorhizal and rhizobial symbioses differ in their regulatory pathways, microsymbiont growth rates, and nodule structure (Ferguson *et al*., 2019; Ardley & Sprent, 2021; Xu & Wang, 2023). Actinorhizal nodules tend to be woodier and larger than rhizobial nodules and may therefore take longer to construct, increasing the time for upregulation. Actinorhizal nodule structure also suggests a lower ability to adjust nodule O_2_ content (Tjepkema, 1988), which, in combination with the greater cost associated with shedding a larger and woodier nodule, may increase the time for downregulation. The lower ability to regulate SNF in actinorhizal symbioses (Andrews *et al*., 2011; Ardley & Sprent, 2021) is supported by reports of actinorhizal N fixers having an obligate SNF strategy (Mead & Preston, 1992; Binkley *et al*., 1994; Menge & Hedin, 2009). However, recent evidence suggests that both actinorhizal and rhizobial N‐fixing trees continue to fix N under excess N supply, at least in some conditions (Taylor & Menge, 2018; Menge *et al*., 2023). Third, temperature, which controls biological activity via its effect on enzyme kinetics, may affect rates of nodule construction, bacterial growth, or response of plants to environmental cues. Thus, time lags may be longer at cold temperatures. Finally, a species' location of origin, which causes adaptation to different environmental cues, may determine how quickly SNF is regulated.

Here, we applied a novel method for measuring SNF and CO_2_ exchange at the whole‐plant scale, in real‐time, continuously, and nondestructively (Bytnerowicz *et al*., 2019) to quantify the time lags in SNF regulation. Specifically, we measured SNF and CO_2_ exchange repeatedly for months as we manipulated N supply to N‐fixing tree seedlings. Species representing tropical and temperate origins and actinorhizal and rhizobial symbioses grew under both warm and cold growing conditions. We then asked: (1) how quickly do plants up‐ and downregulate SNF and (2) how do time lags in the regulation of SNF differ across species that represent temperate vs tropical origins, cold vs warm growing temperatures, and actinorhizal vs rhizobial symbiotic associations? If downregulation takes longer than 1 wk (or even 1 d), this suggests that time lags may play a role in the paradox of sustained N exports from tropical forests, and if time lags are longer in temperate or actinorhizal N fixers, or under cold growing conditions, then time lags may play a role in latitudinal patterns of N fixer abundance and N limitation.

## Materials and Methods

### Experimental setup

We grew four N‐fixing tree species: *Gliricidia sepium* Jacq. Steud. (tropical, rhizobial), *Robinia pseudoacacia* L. (temperate, rhizobial), *Morella cerifera* L. (tropical, actinorhizal), and *Alnus rubra* Bong. (temperate, actinorhizal). Plants were grown from seeds that were surface sterilized before sowing in autoclaved growing medium in germination trays. The growing medium was a crushed granite called Gran‐I‐Grit Starter (North Carolina Granite Corp., NC), which was very low in N and did not bind NO_3_^−^ and NH_4_^+^ (Supporting Information Fig. S1). Seeds were purchased from online vendors (Sheffield's Seed Co. (*A. rubra* and *R. pseudoacacia*; Locke, NY, USA), Rarexoticseeds (*G. sepium*; Montreal, QC, CA), and Bountiful Gardens (*M. cerifera*; Willits, CA, USA)). Upon germination, seedlings were inoculated with a nodule slurry and liquid culture from species‐specific field‐collected nodules (see Notes S1).

Seedlings were fertilized weekly within the germination trays with NH_4_NO_3_ at a rate of 6.6 g N m^−2^ yr^−1^ and N‐free Hoaglands solution (Ross, 1974) at a rate of 0.2 g P m^−2^ yr^−1^, with all other nutrients stoichiometrically balanced to P. All area units in fertilization rates are the surface areas of the pots in which seedlings were grown. After a minimum of 1 month following germination, seedlings were evaluated for SNF activity (see the ‘SNF and carbon exchange measurements’ section). When they were actively fixing N, they were transferred to 1‐l square pots with the same growing medium. Within the 1‐l pots, half the plants were fertilized weekly under a high N treatment (30 g N m^−2^ yr^−1^) and half were fertilized under a low N treatment (1.5 g N m^−2^ yr^−1^). Non‐N nutrients were kept at the same concentration as in the germination trays. These fertilization rates were meant to ensure that plants were N limited at low N and N saturated at high N, following Menge *et al*. (2015). These N supply treatments are high relative to what is encountered by plants in their natural environments, but were chosen to assess how quick plants can change SNF. When we grew these species (and others) in a high‐light glasshouse, there was still some SNF at these high rates of fertilization (Menge *et al*., 2024), but in a lower‐light glasshouse (Menge *et al*., 2024) and the lower‐light growth chambers we used here (to be described later), there was no SNF at these same high rates.

Seedlings were grown under either cold (21°C : 15°C day : night; mean 18.5°C) or warm growing conditions (31°C : 25°C day : night; mean 28.5°C) in Percival PGC‐105 (Perry, IA) temperature‐controlled plant growth chambers. These growing temperatures represent a range of typical temperate and tropical growing season temperatures. Photosynthetic Photon Flux Density during plant growth was 800 μmol m^−2^ s^−1^, the photoperiod was 14 : 10 (day : night), relative humidity was 70%, and CO_2_ was 400 ppm. Water was supplied two to three times per week from the bottom by placing pots in trays (four pots per tray) to ensure that plants were not water limited and so that nutrients were not washed out of pots.

We induced downregulation of SNF by switching the fertilization rate from low N to high N (1.5–30 g N m^−2^ yr^−1^). We induced upregulation by rinsing each pot with 3 l of water (which reduces extractable nutrients to near the low baseline level for this growing medium; Fig. S1) and switching the fertilization rate from high N to low N (30–1.5 g N m^−2^ yr^−1^). Seedlings were grown in their experimental pots until they were roughly similar in size (Table S1) before changing their N supply (6–12 months due to different growth rates across species and treatments). For seedlings switched from high N to low N, plants received N‐free Hoaglands but no N fertilizer on the day growing conditions were changed (Day ‘0’) and both low N and N‐free Hoaglands fertilizers on following weeks. Upon switching fertilization rates, plants were reinoculated with nodule slurry and liquid culture (Notes S1) to ensure plants had ample access to rhizobia or *Frankia*. We measured downregulation in four individual plants per treatment (i.e. species and growing temperature combination) for a total of 32 plants. For upregulation, we increased the starting sample size to six to eight plants per treatment, for a total of 50 plants, because initial measurements showed higher variation across individuals in upregulation and because not all plants successfully upregulated SNF. Several plants that we switched from high to low N did not upregulate SNF, or had SNF rates that were so low that they were removed before analysis, resulting in a final sample size of 33 plants for upregulation (Notes S2).

### SNF and CO_2_ exchange measurements

We measured ethylene and CO_2_ exchange in a closed chamber and at the whole‐plant scale using a Picarro G2106 ethylene analyzer and LI‐COR 6262 CO_2_ and H_2_O analyzer (Bytnerowicz *et al*., 2019). This method (acetylene reduction assays by cavity ring‐down laser absorption spectroscopy (ARACAS); Cassar *et al*., 2012; Bytnerowicz *et al*., 2019) makes real‐time, continuous, and nondestructive measurements of nitrogenase activity, which can be used to quantify how nitrogenase activity changes in response to environmental conditions over timescales that range from seconds to hours (continuous data at 1 Hz, e.g. Bytnerowicz *et al*. (2022); Mifsud *et al*. (2023)) or days to months (repeated measurements, as in this study). ARACAS quantifies ethylene production by nitrogenase in root nodules exposed to acetylene at a rate that is proportional to the rate of SNF (Hardy *et al*., 1968).

In our setup, air was circulated by two explosion‐proof muffin fans (MMF24‐3B Matticks Industries Inc., Houston, TX) within a 40.5‐l incubation chamber made of translucent acrylic. Temperature during measurements was controlled by copper coils surrounding the plant, which were connected to a water bath (Thermo Fisher Scientific Inc., Waltham, MA). SNF and CO_2_ exchange measurements occurred at the daytime growing temperature for all plants (i.e. 21°C and 31°C for plants from the cold and warm growth chambers, respectively). Measurement temperatures were verified via measurements at the surface of the growing medium with an iButton (precision of 0.5°C; Maxim Integrated, San Jose, CA). The incubation chamber for measuring SNF and CO_2_ exchange was placed within the plant growth chambers for all measurements so that measurements were over stable conditions, and so they best replicated daytime growing conditions (e.g. PPFD of 800 μmol m^−2^ s^−1^ within the incubation chamber, equivalent to light levels under which plants were grown).

ARACAS measurements were typically made at the following time points relative to each switch in N supply: immediately before the switch (Day ‘0’), 3 d after the switch (the maximum frequency to not dampen nitrogenase activity; Bytnerowicz *et al*., 2019), and every 1–2 wk after the switch until N fixation rates stabilized. ARACAS measurements on a given day were made before plants were fertilized and, on Day ‘0’, before treatments (high or low N supply) were switched. Each measurement consisted of measuring background rates of plant ethylene production (which was typically negligible) for 10–15 min before injecting acetylene and measuring rates of SNF for 15 min. Net whole‐plant photosynthesis was measured concurrently with nitrogenase activity. After nitrogenase activity was measured, acetylene was cleared from the incubation chamber and whole‐plant respiration was measured in the dark for a minimum of 15 min (adequate for ensuring respiration rates equilibrated).

ARACAS measurements were made at 2% acetylene for three reasons: to reduce the potential negative effects of acetylene and ethylene on the plant or bacteria, to optimize the detection threshold for measuring nitrogenase activity (Bytnerowicz *et al*., 2019), and to increase safety (> 2.5% acetylene is explosive). Acetylene was made in the laboratory from calcium carbide to improve the precision of measurements because the background ethylene concentration in acetylene tanks is 300–750 times greater than in laboratory‐made acetylene, causing the detection threshold to be 75–600 times greater (worse) for tank‐derived acetylene (Bytnerowicz *et al*., 2019). Following Bytnerowicz *et al*. (2019), the rate of nitrogenase activity was proportional to:

(Eqn 1) $$V_{\max}\left(t\right)=\left[\frac{dE\left(t\right)}{dt}+k_{\mathrm{eff}}E\left(t\right)\right]\times \left[\frac{K_{m}}{A\left(0\right)e^{-k_{\mathrm{eff}}t}}+1\right],$$

where $V_{\max}\left(t\right)$ is the substrate‐saturated rate of acetylene reduction, t is time, $\frac{dE\left(t\right)}{dt}$ is the rate of change in ethylene over time, $k_{\mathrm{eff}}$ is the leak rate constant for gas out of the incubation chamber (assumed to be the same for ethylene and acetylene), $E\left(t\right)$ is the ethylene concentration, *K*_m_ is the Michaelis–Menten half‐saturation constant for ethylene production as a function of acetylene, and $A\left(0\right)$ is the starting acetylene concentration. Of these, ethylene and water (used to correct the measured ethylene data which are diluted by rising humidity) were measured in real‐time (frequency of *c*. 1 Hz) by the Picarro G2106. The $k_{\mathrm{eff}}$ and *K*_m_ parameter values were calculated independently (see Bytnerowicz *et al*., 2019, 2022). For our setup, $k_{\mathrm{eff}}$ was 0.0045 (0.0010, 0.0105) hr^−1^ (mean and 95% CI; Bytnerowicz *et al*., 2019). We used species‐specific *K*_m_ values, determined for each species across a range of growing and measurement temperatures (Table S2; Bytnerowicz *et al*., 2022). As detailed in Bytnerowicz *et al*. (2019), we are interested in the substrate‐saturated rate of acetylene reduction because the substrate of interest in the wild is N_2_ gas, which is presumed to be always at saturating levels.

$V_{\max}\left(t\right)$ was calculated from the last 5 min of each incubation in plants that did not experience an acetylene or ethylene induced decline (Minchin *et al*., 1983; all species except *Morella cerifera*). For plants that showed a decline in nitrogenase activity, we fit a 480‐point moving regression to quantify the maximum nitrogenase rate after equilibration and before the decline. Uncertainty was propagated by parametric bootstrapping (Clark, 2007), where the probability density function of $V_{\max}\left(t\right)$ is:

(Eqn 2) $$\begin{array}{c} \mathrm{PDF}\left(V_{\max}\left(t\right)\right)=\left[N\left(\mu_{\frac{dE\left(t\right)}{dt}},\sigma_{\frac{dE\left(t\right)}{dt}}\right)+\Gamma \left(\alpha_{k_{\mathrm{eff}}},\beta_{k_{\mathrm{eff}}}\right)\times E\left(t\right)\right]\\ \times \left[\frac{\Gamma \left(\alpha_{K_{m}},\beta_{K_{m}}\right)}{N\left(\mu_{A\left(0\right)},\sigma_{A\left(0\right)}\right)e^{-t\times \Gamma \left(\alpha_{k_{\mathrm{eff}}},\beta_{k_{\mathrm{eff}}}\right)}}+1\right]. \end{array}$$

Here, $\frac{dE\left(t\right)}{dt}$ and $A\left(0\right)$ follow normal distributions (means μ and SD σ) and $k_{\mathrm{eff}}$ and *K*_m_ follow gamma distributions (rates α and shapes β). Finally, nitrogenase activity rates were converted to N fixation rates (SNF) by using species‐specific conversion factors between ethylene production and SNF quantified in Bytnerowicz *et al*. (2022; Table S3). Converting nitrogenase activity to SNF changes the absolute rates in Figs S2 and S3, but does not affect model parameters responsible for how quickly up‐ or downregulation happen during the course of the experiment (parameters r and $t_{L}$ in Eqn 5, below).

Whole‐plant carbon exchange was estimated following (Bytnerowicz *et al*., 2019) as:

(Eqn 3) $$\begin{array}{c} \mathrm{NPE}\left(t\right)=\left[\frac{d{\mathrm{CO}}_{2}\left(t\right)}{dt}+k_{\mathrm{eff}}\left({\mathrm{CO}}_{2}\left(t\right)-{\mathrm{CO}}_{2}^{A}\right)\right]V, \end{array}$$

where $\mathrm{NPE}\left(t\right)$ is net plant CO_2_ exchange at any point in time, $\frac{d{\mathrm{CO}}_{2}\left(t\right)}{dt}$ is the rate of change of CO_2_ in the incubation chamber, $k_{\mathrm{eff}}$ is the same leak rate constant as for measuring SNF, ${\mathrm{CO}}_{2}\left(t\right)$ is the CO_2_ concentrations at any point in time in the incubation chamber, ${\mathrm{CO}}_{2}^{A}$ is the ambient CO_2_ concentration (assumed to be 400 ppm outside of the incubation chamber), and V is the molar volume of the incubation chamber. Uncertainty in $\mathrm{NPE}\left(t\right)$ was quantified by parametric bootstrapping, with a probability density function of:

(Eqn 4) $$\begin{array}{c} \mathrm{PDF}\left(\mathrm{NPE}\left(t\right)\right)=\left[N\left(\mu_{\frac{d{\mathrm{CO}}_{2}\left(t\right)}{dt}},\sigma_{\frac{d{\mathrm{CO}}_{2}\left(t\right)}{dt}}\right)+\Gamma \left(\alpha_{k_{\mathrm{eff}}},\beta_{k_{\mathrm{eff}}}\right)\times \left({\mathrm{CO}}_{2}\left(t\right)-{\mathrm{CO}}_{2}^{A}\right)\right]\times V, \end{array}$$

where $\frac{d{\mathrm{CO}}_{2}\left(t\right)}{dt}$ has a normal distribution and $k_{\mathrm{eff}}$ has a gamma distribution. Net plant photosynthesis was estimated as $\mathrm{NPE}\left(t\right)$ in the light, whole‐symbiosis respiration was $\mathrm{NPE}\left(t\right)$ in the dark, and apparent photosynthesis was the difference between the two (Wohlfahrt & Gu, 2015). A correction for the CO_2_ concentration was applied to the net and apparent photosynthesis fluxes (see Notes S3; Table S4).

### Statistics

We primarily focused on how long it took SNF to approach a new equilibrium following a drastic increase or decrease in N supplied through fertilizer. Since we measured SNF over the course of several weeks, we divided by whole‐symbiosis respiration to account for changes in plant size over the course of the experiment (but see Figs S4, S5 for SNF not divided by whole‐symbiosis respiration). To quantify how SNF changed as a function of time (*t*), we used a lagged sigmoidal function:

(Eqn 5) $$\begin{array}{c} \mathrm{SNF}\left(t\right)=\frac{K}{1+e^{-r\left(t-t_{L}\right)}}, \end{array}$$

where K is the saturated rate of SNF, r controls the maximum steepness of the curve, and $t_{L}$ is the time until K is halved. If r is positive, SNF is upregulated; if r is negative, SNF is downregulated.

A negative log likelihood function was built for each species and growing temperature using Eqn 5. Preliminary analyses analyzing all plants and growing temperatures together with nonlinear mixed effects models failed to converge. Thus, Eqn 5 was fit for each species and growing temperature using all fixed effects, with separate parameter estimates of K and $t_{L}$ for each individual plant but with r fit at different levels of organization (e.g. one r for all plants, separate r for each symbiotic type, and separate r for each growing temperature; Tables S5–S8). Individual plant $t_{L}$ estimates were extracted from the best model and compared at different levels of organization (analogous to the r analysis described in the previous sentence) with several analysis of variance (ANOVA) models. We also tested for the effects of plant size (using the whole‐symbiosis respiration rate at Day 0) and maximum SNF on the time for up‐ and downregulation of SNF (Notes S4). Eqn 5 was fit with the ‘mle2’ function in the bblme package (Bolker & R Development Core Team, 2023) in R (R Core Team, 2022). The best models (both for r and $t_{L}$) were chosen using finite‐size corrected Akaike information criterion (AIC_c_). In cases where the ΔAIC_c_ between competing models was < 2, suggesting more‐or‐less equivalent models (Burnham & Anderson, 2004), we used a likelihood ratio test to determine whether a more complex model was significantly better at the 95% confidence level. The 95% confidence intervals (CI) of parameters and functions of parameters were estimated by parametric bootstrapping using a normal distribution (Clark, 2007).

## Results

### How quickly do individual plants up‐ and downregulate SNF?

**Fig. 1:**
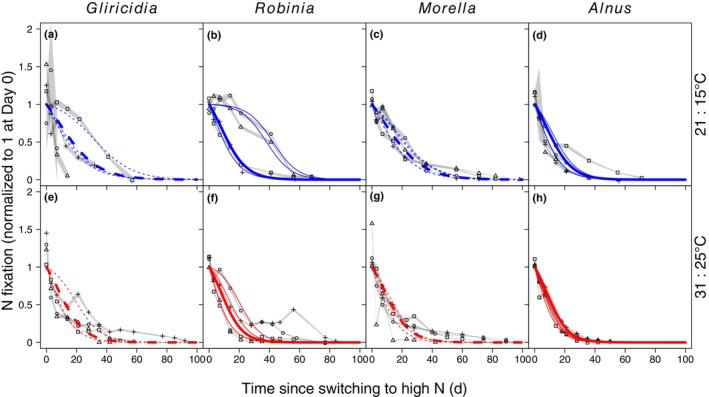
Time‐series data and maximum likelihood fits for downregulation of whole‐symbiosis level nitrogen (N) fixation. (a, e) *Gliricidia sepium* (tropical, rhizobial), (b, f) *Robinia pseudoacacia* (temperate, rhizobial), (c, g) *Morella cerifera* (tropical, actinorhizal), and (d, h) *Alnus rubra* (temperate, actinorhizal) were grown under two temperature regimes: (a–d) 21 and 15°C during the day and night, respectively, and (e–h) 31 and 25°C. Time = 0 is when the plants switched from a low to high N fertilization rate. N fixation at the whole‐symbiosis scale was measured with repeated, nondestructive measurements of individual plants (different symbols for each individual in a panel). Gray shading represents the 95% confidence interval (CI) of each measurement, with upper and lower CIs connected across adjacent time points for each individual via straight lines. N fixation is expressed per whole‐symbiosis respiration rate to account for differences in plant size and then normalized to 1 at Day 0 (see Supporting Information Figs S2, S4 for versions that are not normalized to 1 and not expressed per whole‐symbiosis respiration, respectively). Curve color indicates growing temperature (blue for cold and red for warm). Curve type indicates biome of origin (solid for temperate and dashed for tropical). All curves show the sigmoid shape of Eqn 5, but with different values of parameters fit to different groups of plants. Thin curves show fits for individual plants where the timescale of downregulation ($t_{L}$) is allowed to vary by plant. Thick curves show the best statistical fits (Tables S5, S7) where the timescale of downregulation ($t_{L}$) does not differ between rhizobial and actinorhizal symbioses, cold or warm growing temperatures, or temperate or tropical origin, but where the maximum rate of downregulation (r) differs for all combinations of temperate vs tropical origins and cold vs warm growing temperatures (but not symbiotic type).

**Fig. 2:**
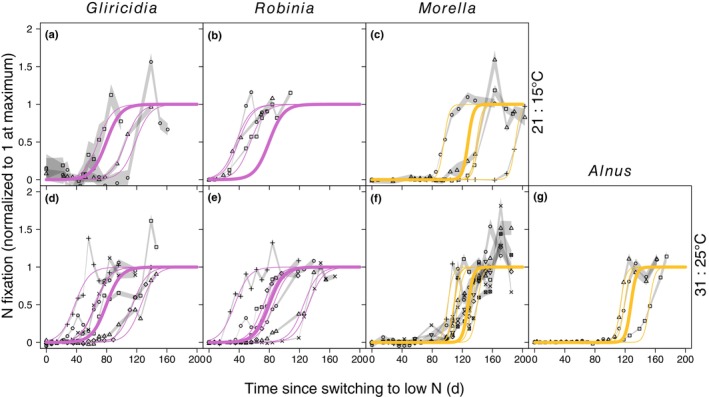
Time‐series data and maximum likelihood fits for upregulation of nitrogen (N) fixation. (a, d) *Gliricidia sepium* (tropical, rhizobial), (b, e) *Robinia pseudoacacia* (temperate, rhizobial), (c, f) *Morella cerifera* (tropical, actinorhizal), and (g) *Alnus rubra* (temperate, actinorhizal), were grown under two temperature regimes: (a–c) 21 and 15°C during the day and night, respectively, and (d–g) 31 and 25°C. Time = 0 is when plants had N rinsed out of the growing medium and were switched from a high to low N fertilization rate. N fixation at the whole‐symbiosis scale was measured with repeated, nondestructive measurements of individual plants (different symbols for each individual in a panel). Gray shading represents the 95% confidence interval (CI) of each measurement, with upper and lower CIs connected across adjacent time points for each individual via straight lines. N fixation is normalized to 1 at the maximum rate (determined by maximum likelihood fits of Eqn 5; see Supporting Information Figs S3 and S5 for versions where N fixation is not normalized to 1 and not expressed per whole‐symbiosis respiration, respectively). Curve color indicates symbiotic type (purple for rhizobial, gold for actinorhizal). All curves show the sigmoid shape of Eqn 5, but with different values of parameters fit to different groups of plants. Thin curves show fits where the timescale of upregulation ($t_{L}$) is allowed to vary by plant and the maximum rate of upregulation (r) differs for rhizobial vs actinorhizal symbioses. Thick curves show the best statistical fits (Tables S6, S8) where both the timescale of upregulation ($t_{L}$) and the maximum rate of upregulation (r) differ across symbiotic type but no other factors.

**Fig. 3:**
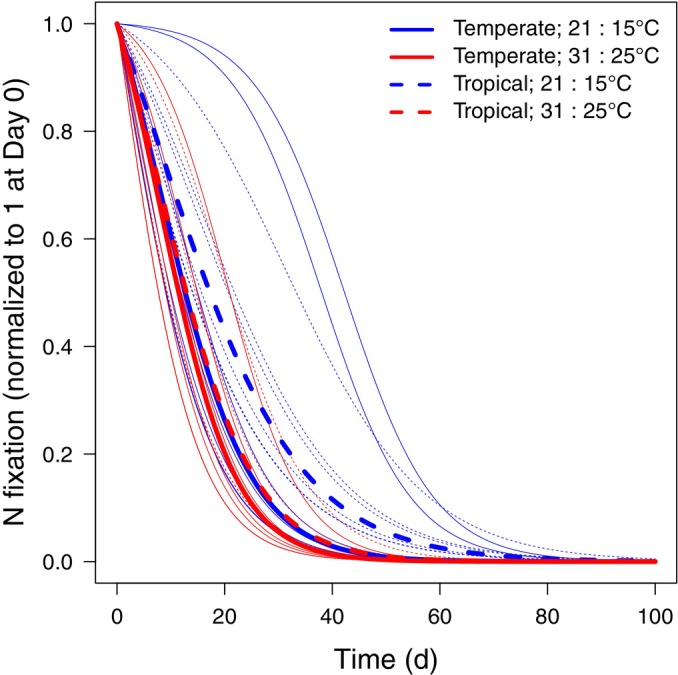
Maximum likelihood fits of downregulation of nitrogen (N) fixation, showing differences due to biome of origin (curve type) and growing temperature (color). These are the same fits shown in Fig. 1. Temperate plants (solid) and plants grown under warmer temperatures (red) downregulate N fixation faster than tropical plants (dashed) and plants grown under colder temperatures (blue). Thin lines represent individual‐plant variation.

**Fig. 4:**
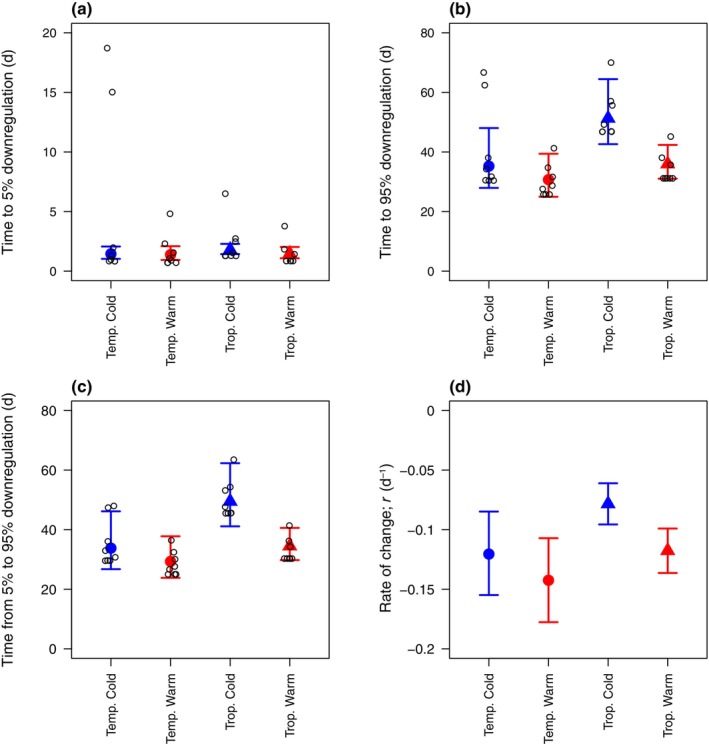
Timescales of downregulation of nitrogen (N) fixation, which were best predicted by an interaction between biome of origin and growing temperature. (a) Time for N fixation to start to decline (5% reduction to 95% of the initial value), (b) time for N fixation to almost completely cease (95% reduction to 5% of the initial value), (c) time from 5 to 95% reduction in N fixation, (d) the r parameter, which primarily controls the maximum rate of change in N fixation. Large blue and red points show model fits with the 95% confidence intervals. Open black points show values for individual plants (corresponding to thin lines in Figs 1, 3). Variation in individual‐plant downregulation from 5 to 95% (c) exists within symbioses × growing temperature bins due to individual‐plant K estimates from Eqn 5 not being equal to N fixation at day = 0.

**Fig. 5:**
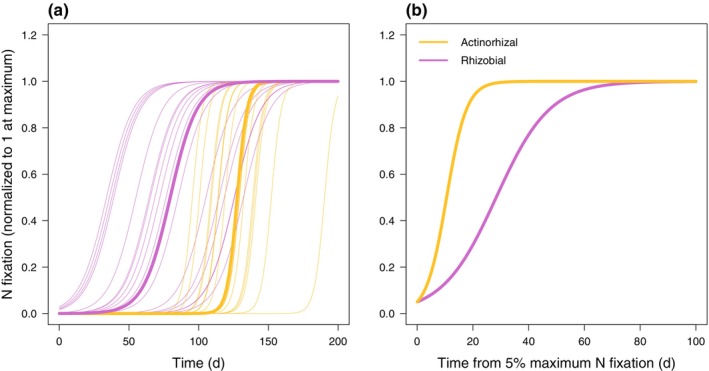
Maximum likelihood fits of upregulation of nitrogen (N) fixation, showing differences due to symbiotic association. (a) Time from when plants had N rinsed out of the growing medium and were switched from a high to low N fertilization rate, (b) time from 5% upregulation of N fixation. Rhizobial plants (purple) initiated N fixation before actinorhizal plants (gold) (a) but had a slower rate of increase in N fixation than actinorhizal plants once N fixation was initiated (b). Thin lines represent individual plants. The curves are the same as in Fig. 2.

**Fig. 6:**
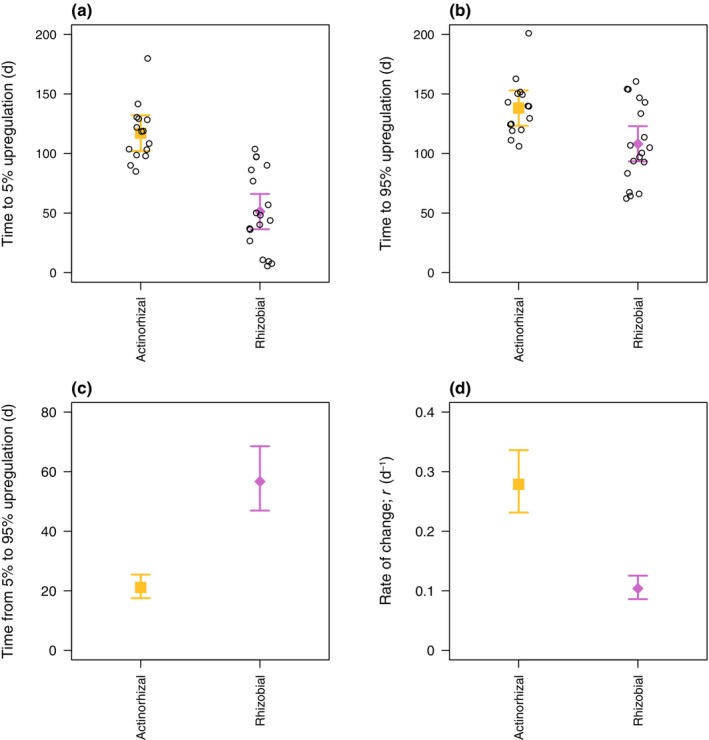
Timescales of upregulation of nitrogen (N) fixation, which were best predicted by symbiotic association. (a) Time for N fixation to start (i.e. reach 5% of maximum N fixation), (b) time for N fixation to approach saturation (i.e. reach 95% of maximum N fixation), (c) time to increase N fixation from 5 to 95% of maximum, (d) the r parameter, which primarily controls the maximum rate of change in N fixation. Large gold and purple points show model fits with the 95% confidence intervals. Open black points show individual plants (corresponding to thin lines in Figs 2, 5).

We tracked changes in SNF in individual plants following a drastic change in N supply (Figs 1, 2, S2–S5). To control for the effect of plant size, all metrics of SNF are expressed relative to whole‐symbiosis respiration unless otherwise indicated. All plants eventually reduced SNF to zero or near zero after they were switched from low to high N supply (Figs 1, 3). Across individual plants, the initial response of SNF to high N supply (5% reduction in SNF, i.e. reduction to 95% of the initial rate) occurred in 1–19 d. The median individual plant took 1 d, and the SD and coefficient of variation (CV) for individual plants was 4 d and 1.5, respectively (Fig. 4a). Reduction of SNF in individual plants to near zero (95% reduction to 5% of the initial rate) took 26–70 d (median 34 d, SD 12 d, CV 0.31) following the switch to high N supply (Fig. 4b). For plants that upregulated SNF (Figs 2, 5), SNF saturated in most plants during the duration of the experiment (see Notes S2 for plants that did not turn on SNF). The time it took for SNF to be detectable at 5% of its maximum rate following a switch to low N supply was highly variable, ranging from 6 to 180 d (median 90 d, SD 44 d, CV 0.54) in individual plants (Fig. 6a). It then took another 21–57 d for SNF to reach 95% of its saturating value (Fig. 6c). The median individual plant took 124 d to reach 95% of its saturating value following the switch to low N supply (SD 33 d, CV 0.27; Fig. 6b). Metrics of plant size are shown in Table S1; raw rates of SNF (unnormalized to whole‐symbiosis respiration) are shown in Figs S4 and S5, and rates of apparent photosynthesis and respiration are shown in Figs S6–S13.

### How do time lags in the downregulation of SNF differ across temperate vs tropical origins, cold vs warm growing temperatures, and actinorhizal vs rhizobial symbioses?

According to the best‐fit model, time lags in downregulating SNF differed by biome of origin, growing temperature, and their interaction (Table S5), driven by variation in the parameter controlling the steepness of the curve in Eqn 5 (r; Fig. 4d) rather than the time to reach half the maximum value ($t_{L}$). Temperate plants (*Alnus* and *Robinia*) and plants grown at warmer temperatures downregulated SNF faster than tropical plants (*Morella* and *Gliricidia*) and plants grown at cooler temperatures (Figs 3, 4). However, tropical plants grown at warmer temperatures downregulated SNF at almost the same rate as temperate plants grown at cooler temperatures (Figs 3, 4). Specifically, temperate plants took 35 (28–48) d (mean and 95% CI) at colder temperatures and 31 (25–39) d at warmer temperatures to downregulate SNF by 95% of their N‐limited rate (Fig. 4b). Tropical plants took 51 (43–64) d at colder temperatures and 36 (31–42) d at warmer temperatures.

### How do time lags in the upregulation of SNF differ across temperate vs tropical origins, cold vs warm growing temperatures, and actinorhizal vs rhizobial symbioses?

Time lags in upregulating SNF were best explained by a model that distinguished by symbiotic association (Table S6), resulting from variation both in the parameter controlling the steepness of the curve in Eqn 5 (r; Fig. 6d) and in $t_{L}$. Rhizobial plants started fixing N much faster than actinorhizal plants (mean and 95% CIs of 51 (36–66) and 117 (102–132) d, respectively, for SNF to reach 5% of its maximum; Fig. 6a). However, once SNF started, actinorhizal plants upregulated SNF much faster than rhizobial plants (mean and 95% CI of 21 (18–25) and 57 (47–69) d, respectively, for SNF to increase from 5 to 95% of its maximum; Fig. 6c). This tradeoff resulted in a reduced difference between rhizobial and actinorhizal plants in how quickly SNF reached 95% of its maximum rate from the time N supply changed (mean and 95% CI of 108 (93–123) and 138 (123–153) d, respectively; Fig. 6b). There was also a marginally significant positive effect of growing temperature on the rate of upregulation (*P* = 0.051), which increased r, the parameter controlling the steepness of Eqn 5.

### Effects of plant size and maximum SNF

In plants that downregulated SNF, there was a weak positive relationship between $t_{L}$ and plant size, both across species (*P* = 0.038) and for *Alnus* at cooler temperatures (*P* = 0.048; Notes S4; Fig. S14). This size effect was minimal, ranging from 0.1 to 0.4 d for the smallest (*Gliricidia* at cooler temperatures) and largest (*Alnus* at warmer temperatures) species × temperature combinations (Table S1; Fig. S14). There was no effect of maximum SNF on downregulation. In plants that upregulated SNF, neither plant size nor maximum SNF influenced upregulation (Notes S4). We did not have the statistical power to assess how plant size or maximum SNF influenced r (Notes S4).

## Discussion

Following a drastic change in soil N supply, it took weeks to months for SNF to adjust to its new rate. When tree seedlings with low soil N supply were switched to a high, more than ample level of soil N supply, it took an average of 31–51 d to downregulate to near zero. Temperate species and seedlings grown in warmer growth chambers downregulated somewhat faster than tropical species and those grown in colder growth chambers, but the timescale was on the order of weeks for all species and growth temperatures. In the other direction, upregulation to near maximum rates took an average of 108–138 d from when N supply was reduced and an average of 21–57 d from when SNF started. Rhizobial symbioses started fixing much sooner than actinorhizal symbioses, but once SNF started, actinorhizal symbioses ramped up faster than rhizobial symbioses. For both symbiotic types, though, the timescale of upregulation was months from the switch and weeks from initialization of SNF. These measurements are the first to our knowledge to quantify the timing of the regulation of SNF at this level of detail.

### The latitudinal gradient in N‐fixing tree abundance

Our results do not resolve the latitudinal gradient in the abundance of N‐fixing trees. Longer time lags reduce the competitiveness of facultative SNF (Menge *et al*., 2009), and facultative SNF can help maintain a high abundance of N‐fixing trees on a landscape (Menge *et al*., 2014), but to explain the latitudinal gradient in the abundance of N‐fixing trees, time lags would need to be longer at colder temperatures, in N fixers from high latitudes, or in actinorhizal N fixers (which dominate the N‐fixing tree community at higher latitudes; Menge *et al*., 2014, 2017). However, the evidence supporting this is mixed. We start by noting that we only studied two temperate, two tropical, two rhizobial, and two actinorhizal species, each at two growing temperatures. With that caveat, we did observe slower downregulation in plants grown at cold temperatures, but temperate species downregulated SNF faster than tropical species. The time for downregulation was similar between tropical species grown at warm temperatures and temperate species grown at cold temperatures, suggesting that downregulation might be similar between tropical and temperate species in their climate of origin. As for upregulation, actinorhizal species did take a longer time overall to turn on SNF, supportive of a mechanism driving potential differences in SNF regulation strategies across latitude. However, the time for upregulation following initiation of SNF may be ecologically more relevant than the time for SNF to upregulate from zero SNF. This is because our high N treatment supplied a much higher rate of N than typical for natural ecosystems and because N‐fixing trees have been shown to maintain some SNF even under excess N supply in some conditions (Taylor & Menge, 2018; Menge *et al*., 2023). Actinorhizal symbioses turned on SNF faster once SNF started. The difference in timescales between up‐ and downregulation also becomes similar when compared in plants that are already fixing N (depending on symbiotic association, growing temperature, and biome of origin, SNF is downregulated from 5 to 95% after 29–50 d on average and is upregulated from 5 to 95% after 21–57 d on average).

### Factors affecting SNF regulation

Understanding the timescale of SNF upregulation depends on the consideration of plant N storage and differences between actinorhizal and rhizobial symbioses relevant to SNF regulation. Growing plants under high N likely results in luxury consumption (Chapin, 1980) of N, which may determine when SNF is initiated if SNF regulation is controlled by internal nutrient stores rather than external N supply (nutrient stores would negate an immediate need for SNF following a reduction in N supply). Although some of our plants experienced a reduction in whole‐plant photosynthesis following a change to low N supply, many did not, suggesting that internal stores of N were sufficient to maintain, and in some cases increase, whole‐plant photosynthesis between the time N supply was reduced and SNF was initiated (Figs S6, S7). We did not see a clear difference between rhizobial and actinorhizal species in how well they were able to maintain photosynthetic rates following a reduction in N supply (which are in part determined by leaf N concentrations; Bytnerowicz *et al*., 2023), suggesting that N storage may not have differed as a function of symbiotic type in our experiment. However, luxury consumption is not the only determinant of when SNF starts, as the initiation of SNF requires successful bacterial infection of roots and time to build functional nodules (Pawlowski & Bisseling, 1996; Gage, 2004). Actinorhizal and rhizobial symbioses differ in the processes controlling nodule development and nodule structure, and in the growth rates of their microsymbionts (slower for *Frankia*; Ferguson *et al*., 2019; Ardley & Sprent, 2021; Xu & Wang, 2023). The mechanisms by which legumes regulate SNF have been well documented, including local and systemic regulation via the autoregulation of nodulation (AON) pathway as well as nitrate regulatory networks (Jeudy *et al*., 2010; Nishida *et al*., 2018; Ferguson *et al*., 2019; Ardley & Sprent, 2021). As a result, rhizobial symbioses can respond to both internal and external nutrient availability by regulating nodule number through controls on rhizobial infection of roots and the development and functioning of existing nodules. Actinorhizal symbioses can also respond to internal and external nutrient cues (Baker *et al*., 1997; Wall *et al*., 2003), for example via the inhibition of nodule activity and root hair development by nitrate (Huss‐Danell, 1997), but the underlying pathways are still poorly described (Xu & Wang, 2023). Furthermore, there is a transient window of susceptibility for nodulation in actinorhizal symbioses, where nodule formation typically happens at the tap root tip at the moment of inoculation (typically leading to clustered nodules; Wall & Huss‐Danell, 1997; Valverde & Wall, 1999). This transient window for nodulation could have played a role in some of our plants not upregulating SNF (e.g. *Alnus rubra* at the cold growing temperature). Our data are consistent with the time to build functional nodules being longer in actinorhizal symbioses, which can be caused by differences across any of these processes.

Faster SNF upregulation in actinorhizal species, once functional nodules were present, may have multiple explanations. First, rhizobial fixers may tune SNF more finely, increasing SNF slowly as internal stores are depleted, while actinorhizal fixers may not initiate SNF until internal stores are depleted, resulting in a higher SNF equilibrium once SNF starts and a need to increase SNF rapidly. A second, and not mutually exclusive, explanation is due to differences in nodule morphology (Pawlowski & Bisseling, 1996; Ardley & Sprent, 2021; Xu & Wang, 2023). Rhizobial fixers may have to build many nodules to increase SNF to the equilibrium rate while an actinorhizal fixer may only need to increase the bacterial population and size of existing nodules to increase SNF (which, despite the relatively slow growth rate of *Frankia*, may be faster than constructing entirely new nodules).

Given that some of the presumed drivers of time lags, such as N storage, scale nonlinearly with plant size (Reich *et al*., 2006; Sardans & Peñuelas, 2015), we tested the possibility that time lags vary with plant size or the realized (for downregulation) or the potential (for upregulation) SNF rate (Notes S4). We found one relationship – larger plants took a longer time to downregulate SNF than smaller plants – although the relationship was weak. However, we note that our plants did not vary widely in size, so a separate experiment across a wider range of plant size and N demand would be necessary to evaluate the effect of size conclusively. Unfortunately, at present there is no clear way to design such an experiment due to the lack of available methods for making repeated SNF measurements in large trees (Soper *et al*., 2021).

Other environmental conditions could affect the regulation of SNF, although we could not evaluate the role of any environmental factors other than growing temperature. The same four species we studied here formed nodules and fixed N at these same high fertilization levels when grown in a high‐light glasshouse (Menge *et al*., 2024), even though they turned fixation off completely in the growth chamber conditions in which we grew them here. Glasshouse and field evidence for complete downregulation of SNF in other contexts is mixed (Binkley *et al*., 1994; Menge & Hedin, 2009; Barron *et al*., 2011; Batterman *et al*., 2013b; Dovrat *et al*., 2018, 2020; Taylor & Menge, 2018; McCulloch & Porder, 2021; Menge *et al*., 2023). In many field conditions, many of which are high light, many species, including some of the same species we studied here (*A. rubra*, *R. pseudoacacia*, and *G. sepium*) continue fixing at relatively high levels when N limitation is presumably alleviated (Binkley *et al*., 1994; Menge *et al*., 2023).

### Ecosystem N losses and competitive ability of facultative N fixers

Our time lag measurements are the first to empirically evaluate theory on the consequences of time lags in regulating SNF (Menge *et al*., 2009). Menge *et al*. (2009) considered two versions of the regulation of SNF in a simple ecosystem model. In both versions, the time lags associated with downregulation and upregulation were the same. The first version assumed that plants perfectly matched SNF to the deficit of supply and demand at a fixed time in the past (the time lag, e.g. 1 d ago). The second version assumed that plants perfectly matched SNF to the deficit of supply and demand over a range of time from the more distant past (e.g. the weeks before a day ago) until that fixed time (e.g. 1 d ago), weighting the more recent past more. Both of these definitions of time lag differ slightly from our estimates based on the time to 95% regulation, but in a conservative direction: if downregulation to 100% in the model takes a day, then downregulation to 95% would take less than a day. The two versions of the model likely bracket the reality of our experiment. The first version implicitly assumes no internal stores of N, which is unrealistic. The time integration in the second version was meant to simulate internal plant storage of N, but it was parameterized with a mature tree in mind rather than a seedling. Thus, we compare our empirical results to both versions of the model. In the first version of the model, time lags > 2 d meant that facultative fixers lost in competition to obligate fixers. In the second version, facultative fixers were relatively more competitive, and time lags needed to be > 2–3 months for the obligate strategy to win (Menge *et al*., 2009). Time lags of more than 1 d led to losses of available N on the order of tens of kg N ha^−1^ yr^−1^ for the first version of the model and hundreds of kg N ha^−1^ yr^−1^ for the second version. Thus, the time lags that we observed – weeks for downregulation and weeks‐months for upregulation – might be long enough to significantly reduce the competitiveness of facultative SNF and are clearly long enough to cause high losses of available N where facultative N fixers are abundant.

N fixers are not always abundant, however, so the abundance of N‐fixing trees must be considered in concert with the dynamics of SNF regulation to understand how time lags help explain high losses of available N at the ecosystem scale (Hedin *et al*., 2009). High losses of available N are characteristic of N saturation, which, according to Hedin *et al*. (2009), suggests a paradox when trying to reconcile them with the downregulation of SNF at the individual‐plant scale under high available N. N‐fixing trees comprise 10–20% of basal area in tropical forests (ter Steege *et al*., 2006; Menge *et al*., 2017, 2019; Gei *et al*., 2018; Steidinger *et al*., 2019), although there can be considerable variation in N‐fixing tree abundance across sites. Loss rates in the model of Menge *et al*. (2009) assumed 100% N fixer abundance and should therefore be adjusted for N‐fixing tree abundance at the scale of watersheds generating high available N losses. For example, if we scale available N loss rates to 10% N‐fixing tree abundance, a 1‐wk lag leads to losses of *c*. 5 kg N ha^−1^ yr^−1^ (according to the first version of the model) or tens of kg N ha^−1^ yr^−1^ (for the second version of the model; Menge *et al*., 2009). These fluxes are similar to common available N hydrological loss rates of 5–10 kg N ha^−1^ yr^−1^ throughout tropical forests (Hedin *et al*., 2003; Brookshire *et al*., 2012). One week to completely downregulate SNF is much faster than the 31–51 d that it took SNF to be downregulated by 95% in our experiment. Additionally, N fixers often account for > 10% tree basal area (Gei *et al*., 2018) and at times achieve dominance at local scales (Bytnerowicz & Menge, 2021). Thus, time lags in regulating SNF can provide a resolution to the paradox of N saturation in tropical forests (Hedin *et al*., 2009).

According to theory, intrinsic regulatory delays in SNF can amplify oscillations between ecosystem N saturation and limitation (Menge *et al*., 2009), but external factors – transient periods of N saturation and limitation from nutrient pulses induced by fluctuations in environmental or ecological conditions – can help initiate the oscillations. These external factors could include drying or rewetting in tropical forests (Lodge *et al*., 1994), which can lead to nutrient release from dead microbial biomass (Singh *et al*., 1989) or epiphytes (Coxson *et al*., 1992), as well as the coupling of water pulses to nutrient cycling in arid and semiarid ecosystems (Austin *et al*., 2004). Disturbance events, such as hurricane‐induced litterfall (Ostertag *et al*., 2003) or insect outbreaks (Yang, 2004), as well as animal excretions (Clark *et al*., 2005), can also create nutrient pulses at multiple spatial scales. The result of these pulses is a mismatch between nutrient mineralization and nutrient uptake, which can induce transient periods of sufficient or insufficient nutrient supply, which lags in SNF can amplify. Our study shows that the lags in SNF are sufficiently large to cause this amplification, and thus to sustain oscillations in N supply regardless of their initial cause.

### Conclusions

We show that time lags in regulating SNF are possibly long enough to hinder the competitive benefit of facultative SNF and are clearly long enough to cause high losses of plant‐available N (Menge *et al*., 2009), resolving the tropical N paradox (Hedin *et al*., 2009). Although time lags were affected by temperature, symbiotic association, and biome of origin, they were not consistent across these explanatory variables in a manner that would imply that they are responsible for a change in SNF regulation and gradient in N‐fixing tree abundance across latitude (Menge *et al*., 2014). Thus, there are likely other explanations for the latitudinal gradient in N‐fixing tree abundance (Houlton *et al*., 2008; Menge *et al*., 2014, 2017; Sheffer *et al*., 2015; Bytnerowicz *et al*., 2022).

## Competing interests

None declared.

## Author contributions

TAB, KLG and DNLM designed the research. TAB performed the research, analyzed the data, and wrote the first draft of the manuscript. TAB, KLG and DNLM edited the manuscript.

## Disclaimer

The New Phytologist Foundation remains neutral with regard to jurisdictional claims in maps and in any institutional affiliations.

## Supporting information


**Fig. S1** Rinse test demonstrating that the growing medium does not bind NH_4_^+^ and NO_3_^−^ tightly.
**Fig. S2** Downregulation of N fixation as a function of time since switching to high N supply (N fixation divided by whole‐symbiosis respiration; absolute).
**Fig. S3** Upregulation of N fixation as a function of time since switching to low N supply (N fixation divided by whole‐symbiosis respiration; absolute).
**Fig. S4** Downregulation of N fixation as a function of time since switching to high N supply (N fixation not divided by whole‐symbiosis respiration; absolute rates).
**Fig. S5** Upregulation of N fixation as a function of time since switching to low N supply N fixation not divided by whole‐symbiosis respiration (absolute rates).
**Fig. S6** Apparent whole‐plant photosynthesis following a switch of plants to high N supply (normalized).
**Fig. S7** Apparent whole‐plant photosynthesis following a switch of plants to high N supply (absolute).
**Fig. S8** Apparent whole‐plant photosynthesis following a switch of plants to low N supply (normalized).
**Fig. S9** Apparent whole‐plant photosynthesis following a switch of plants to low N supply (absolute).
**Fig. S10** Whole‐symbiosis respiration following a switch of plants to high N supply (normalized).
**Fig. S11** Whole‐symbiosis respiration following a switch of plants to high N supply (absolute).
**Fig. S12** Whole‐symbiosis respiration following a switch of plants to low N supply (normalized).
**Fig. S13** Whole‐symbiosis respiration following a switch of plants to low N supply (absolute).
**Fig. S14** The *t*L parameter from Eqn 5 as a function of the whole‐symbiosis respiration rate in plants downregulating SNF.
**Notes S1** Inoculum details.
**Notes S2** Plants that failed to upregulate SNF.
**Notes S3** CO_2_ corrections for photosynthetic calculations.
**Notes S4** Effects of plant size and maximum SNF on up‐ and downregulation of SNF.
**Table S1** Size of plants at Day 0.
**Table S2** Michaelis–Menten half‐saturation constants (*K*_m_) for measuring nitrogenase activity with acetylene.
**Table S3** Conversion factors for paired ARACAS and ^15^N_2_ incubations on severed nodules.
**Table S4** Parameter values for best‐fit models of Eqn S1 for apparent photosynthesis as a function of CO_2_.
**Table S5** ΔAIC_c_ values for downregulation of N fixation.
**Table S6** ΔAIC_c_ values for upregulation of N fixation.
**Table S7** Parameter values for best‐fit model of Eqn 5 for downregulation of SNF.
**Table S8** Parameter values for best‐fit model of Eqn 5 for upregulation of SNF.
Please note: Wiley is not responsible for the content or functionality of any Supporting Information supplied by the authors. Any queries (other than missing material) should be directed to the *New Phytologist* Central Office.

## Data Availability

Data and R code are available on Zenodo: https://doi.org/10.5281/zenodo.15530795 (Bytnerowicz *et al*., 2025) and on GitHub: https://github.com/tbytnero/Bytnerowicz_Griffin_Menge_2025_NewPhytologist.
