# Supplementary material for: Time lags in the regulation of symbiotic nitrogen fixation
Source: New Phytol. 2025 Jun 11;247(4):1680–93. doi: 10.1111/nph.70295 (PMC12267930; doi:10.1111/nph.70295)
Supplement: Supplementary file 1 — Fig. S1 Rinse test demonstrating that the growing medium does not bind NH4 + and NO3 − tightly. Fig. S2 Downregulation of N fixation as a function of time since switching to high N supply (N fixation divided by whole‐symbiosis respiration; absolute). Fig. S3 Upregulation of N fixation as a function of time since switching to low N supply (N fixation divided by whole‐symbiosis respiration; absolute). Fig. S4 Downregulation of N fixation as a function of time since switching to high N supply (N fixation not divided by whole‐symbiosis respiration; absolute rates). Fig. S5 Upregulation of N fixation as a function of time since switching to low N supply N fixation not divided by whole‐symbiosis respiration (absolute rates). Fig. S6 Apparent whole‐plant photosynthesis following a switch of plants to high N supply (normalized). Fig. S7 Apparent whole‐plant photosynthesis following a switch of plants to high N supply (absolute). Fig. S8 Apparent whole‐plant photosynthesis following a switch of plants to low N supply (normalized). Fig. S9 Apparent whole‐plant photosynthesis following a switch of plants to low N supply (absolute). Fig. S10 Whole‐symbiosis respiration following a switch of plants to high N supply (normalized). Fig. S11 Whole‐symbiosis respiration following a switch of plants to high N supply (absolute). Fig. S12 Whole‐symbiosis respiration following a switch of plants to low N supply (normalized). Fig. S13 Whole‐symbiosis respiration following a switch of plants to low N supply (absolute). Fig. S14 The tL parameter from Eqn 5 as a function of the whole‐symbiosis respiration rate in plants downregulating SNF. Notes S1 Inoculum details. Notes S2 Plants that failed to upregulate SNF. Notes S3 CO2 corrections for photosynthetic calculations. Notes S4 Effects of plant size and maximum SNF on up‐ and downregulation of SNF. Table S1 Size of plants at Day 0. Table S2 Michaelis–Menten half‐saturation constants (K m) for measuring nitrogenase activity with acetylene. Ta [file NPH-247-1680-s001.pdf]

New Phytologist

***New Phytologist* Supporting Information**

Article title: Time lags in the regulation of symbiotic nitrogen fixation
Authors: Thomas A. Bytnerowicz, Kevin L. Griffin, & Duncan N.L. Menge
Article acceptance date: 23 May 2025

The following Supporting Information is available for this article:

Notes S1 | Inoculum details. Species-specific nodules were collected from the field from Florida, Oregon, and NY (see Bytnerowicz *et al*., 2022 for more details on seed and nodule sources). Upon collection from the field, crushed nodules were used to inoculate a subset of seedlings. These seedlings were then grown to supply nodules for the experiment and to prepare inoculum. Nodules were surface sterilized by vortexing for 60 seconds in a bleach/Contrex lab detergent mix. Post surface-sterilization, nodules were vortexed six times at 20 seconds in deionized water. Nodules were then crushed with a sterile scalpel and cultured on ten yeast mannitol agar plates for each species. Colonies were re-plated three times before they were placed into a yeast mannitol liquid media in culture tubes for one week on a shaker. Liquid media from culture tubes were then combined and plate counts were conducted so that inoculum could be diluted with sterile yeast mannitol liquid media to 5×10^8 cells mL^{-1} for cryopreservation. Inoculum was prepared for cryopreservation by mixing the liquid culture with 30% glycerol and 30% deionized water, placing the mixture in micro-centrifuge tubes, vortexing, and flash-freezing with liquid nitrogen. Following flash-freezing, samples were held in a –30 °C freezer until they were ready to use. The preserved liquid culture was removed from the freezer 2–4 hours prior to inoculating plants to thaw and was diluted to 10^8 cells mL^{-1}. We then added 1 mL, containing 10^8 cells, to each seedling. In addition to inoculating plants with the liquid culture, we prepared a nodule slurry for inoculating plants by surface-sterilizing ~5 mL of fresh nodules, crushing the surface-sterilized nodules with a scalpel, diluting the slurry to 15 mL with deionized water and adding 1 mL per seedling. We used this approach to ensure that seedlings had a saturating number of N-fixing bacteria (liquid culture) and that experimental plants were exposed to a diverse range of bacteria found within nodules in the field (nodule slurry).

Notes S2 | Plants that failed to up-regulate SNF. At 21/15 °C, 1 out of 6 *M. cerifera* plants, 6 out of 6 *A. rubra* plants (one of which died), 3 out of 6 *G. sepium* plants, and 1 out of 6 *R. pseudoacacia* plants did not fix N during the time period SNF was measured. At 31/25 °C, 3 out of 6 *A. rubra* plants (two of which died) did not fix N during the period of measurement. Plants that never fixed N were excluded from analyses because our analyses required having non-zero SNF rates. Additionally, 1 *M. cerifera* and 2 *R. pseudoacacia* plants at 21/15 °C and 1 out of the 4 *A. rubra* plants at 31/25 °C that up-regulated SNF had such low rates that they were excluded from analysis. This resulted in sample sizes of 4, 0, 3, and 3 for *M. cerifera*, *A. rubra*, *G. sepium*, and *R. pseudoacacia* at 21:15 °C and 8, 3, 6, 6 at 31:25 °C, respectively. We suspect that the lack of up-regulation in these plants resulted from inadequate access to symbiotic bacteria at the

time when up-regulation would have been beneficial, which was typically months after the last time we had inoculated them. However, we cannot discount the possibility that the plants did not up-regulate SNF for some other reason (e.g., the transient window of susceptibility for nodulation in actinorhizal symbioses, as discussed in the main text).

Notes S3 | CO_2 corrections for photosynthesis calculations. Following Bytnerowicz *et al.*, 2019, we used a LI-COR 6800 photosynthesis measurement system to make CO_2 correction curves for our whole-plant apparent photosynthesis measurements. This step was necessary because our whole-symbiosis closed chamber resulted in changing CO_2 concentrations during the course of measurement, which affected apparent rates of photosynthesis. With the LI-COR 6800 we made leaf-level photosynthesis measurements at reference CO_2 values of 250–800 ppm, a VPD of 1.2 kPa, and PPFD of 800 µmol m^{-2} s^{-1} (this PPFD and VPD are analogous to what plants are exposed to in the ARACAS chamber). Leaf temperature was set to either 21 or 31°C, depending on whether the measurement was for a plant from the cold or warm growth chamber, respectively. Measurements were logged once stomatal conductance and CO_2 assimilation rates equilibrated. The timescale that we used (allowing for the equilibration of stomatal conductance and/or RuBisCO enzyme deactivation) is slower than that used for estimating J_{max} and Vc_{max} values via an A/C_i curve. We used this timescale because we applied the correction to apparent photosynthesis rates in the ARACAS chamber, where CO_2 concentrations changed relatively slowly (due to the large size of the ARACAS chamber relative to most plants). We made one CO_2 response curve per species/growing temperature combination. A shifted Michaelis-Menten curve was used to convert apparent photosynthesis values measured at chamber CO_2 concentrations to what would be expected at 400 ppm:

$$Apparent\ Photosynthesis = \frac{a \times (CO_2 - c)}{b + (CO_2 - c)}, \qquad (S1)$$

where a is the CO_2-saturated apparent photosynthesis rate, $b + c$ is the half-saturation constant (the CO_2 concentration where apparent photosynthesis is half of a) and c is the shift of the x-intercept (the CO_2 concentration where apparent photosynthesis is zero). A negative log likelihood function was built for each species. One version had allowed parameters b and c to vary by growing temperature and another did not allow b and c to vary by growing temperature. Eq. S1 was fit with the “mle2” function in the “bblme” package (Bolker & R Core Team, 2014) in R (R Core Team 2022). The best model for each species (i.e., temperature effect or no temperature effect) was determined by AIC_c. The best model for *A. rubra* and *M. cerifera* did not include a temperature effect (ΔAIC_c = 2.4 and 7.8, respectively), while the best model for *G. sepium* and *R. pseudoacacia* did include a temperature effect (ΔAIC_c = 10.5 and 67.7, respectively). See Table S4 for fitted values of a, b, and c for each species and temperature.

Notes S4 | Effects of plant size and maximum SNF on up- and down-regulation of SNF. We attempted to test for the effects of plant size, measured as whole-symbiosis respiration rate at day 0, and maximum SNF (SNF normalized by whole-symbiosis respiration) on r and t_L. We were able to do this successfully for t_L but did not have the statistical power to do this for r. As described in the main text, t_L was estimated for individual plants while r was estimated at

different levels of organization, starting at the treatment level (unique species x temperature combinations, e.g., *Alnus* at the cold growing temperature). Thus, the approach we took for *r* was to make *r* a linear function of plant size or maximum SNF in our negative log likelihood functions. However, the optimizer (mle2) was unable to invert the observed-information (Hessian) matrix at the solution. This could be due to a combination of too few datapoints (272 data points split across 32 plants for down-regulation and 515 datapoints split across 33 plants for up-regulation) and too little variation in plant size (Table S1).

For t_L, we used post-hoc analyses to estimate whether t_L varied as a function of all combinations of plant size, maximum SNF, and the predictors described in the main text (e.g., temperature, symbiotic type, biome). We did this both within individual treatments and across all treatments. For down-regulation, we log-transformed t_L prior to our analyses (due to many t_L estimates being near zero and a few being much larger). Across all treatments, the best model for down-regulation included a size effect, where t_L was greater in larger plants ($p = 0.038$; Fig. S14). At the treatment level, there was only a clear relationship (at the 95% confidence level; also positive) for *Alnus* at the cold growing temperature ($p = 0.048$). Because the significance of these relationships is weak and because plant size did not vary dramatically across plants (Table S1), we do not include plant size as a predictor of the time for down-regulation when it is discussed in the main text. The size effect on time scales of down-regulation is also minimal, as median whole-symbiosis respiration rates for the biome-temperature bins ranged from 20.1 to 30.5 nmol CO_2 s^{-1}, which translate to t_L estimates ranging from 0.1 to 0.4 days (Fig. S14). Fig. S14 also demonstrates that plant size does not explain the outliers in Fig. 4a,b. For example, the few individuals that took much longer to down-regulate SNF by 5% (points above 95% confidence intervals in Fig. 4a) did not have particularly high whole-symbiosis respiration rates and were outside of the 95% confidence interval in Fig. S14. As for up-regulation, the lowest AIC_c was for t_L depending on both symbiotic association and maximum SNF, followed by t_L depending solely on symbiotic association (delta $AIC_c = 0.5$). In the model with t_L depending on both symbiotic association and maximum SNF, t_L decreased with maximum SNF, but the effect was only marginally significant ($p = 0.075$). Within treatments, there was no effect of size or maximum SNF on up-regulation. Thus, in the main text we use the model that only includes symbiotic association as a predictor for t_L.

Table S1 | Size of plants at day zero. Means and standard deviations of three measures of plant size at the time plants were switched from either low to high N supply (down-regulation) or high to low N supply (up-regulation). The measures are height (measured to the tallest leaf), diameter at base, and whole-symbiosis respiration rate. Data are for plants that were included in the analyses in the main text.

Plant Species	Growing Temperature	Height (St. Dev.) [cm]	Diameter at Base (St. Dev.) [mm]	Whole-symbiosis Respiration (St. Dev) [nmol CO_2 s^{-1}]
		Down-regulation		
G. sepium	21/15 °C	7.5 (1.2)	10.9 (3.7)	16.1 (7.1)
G. sepium	31/25 °C	10.9 (0.3)	8.5 (0.7)	20.2 (2.8)
R. pseudoacacia	21/15 °C	14.8 (5.9)	5.8 (1.9)	24.9 (8.5)
R. pseudoacacia	31/25 °C	15.5 (3.3)	4.1 (0.5)	26.1 (6.1)
M. cerifera	21/15 °C	21.7 (4.0)	4.6 (1.7)	31.1 (11.7)
M. cerifera	31/25 °C	17.2 (4.9)	2.7 (0.7)	25.5 (15.3)
A. rubra	21/15 °C	13.4 (1.8)	6.7 (1.5)	34.8 (15.4)
A. rubra	31/25 °C	22.5 (3.5)	5.1 (0.6)	38.4 (8.9)
		Up-regulation		
G. sepium	21/15 °C	6.7 (2.0)	7.8 (6.2)	10.1 (4.2)
G. sepium	31/25 °C	11.6 (3.6)	5.8 (1.7)	13.6 (2.8)
R. pseudoacacia	21/15 °C	18.8 (4.5)	6.2 (1.3)	28.0 (2.4)
R. pseudoacacia	31/25 °C	17.3 (4.4)	4.1 (0.6)	17.9 (2.8)
M. cerifera	21/15 °C	19.4 (2.3)	4.6 (0.2)	23.4 (0.1)
M. cerifera	31/25 °C	12.4 (1.9)	2.1 (0.5)	12.3 (2.2)
A. rubra	21/15 °C	NA	NA	NA
A. rubra	31/25 °C	26.1 (7.6)	4.6 (1.0)	20.7 (6.6)

Table S2 | Michaelis–Menten half-saturation constants (K_m) for measuring nitrogenase activity with acetylene (in units of % acetylene). This table can also be found in Bytnerowicz *et al*. (2022) *Nature Plants*, Supplementary Table 4.

Plant Species	K_m (95% CI) [%]
Morella cerifera	2.13 (1.91, 2.37)
Alnus rubra	1.65 (1.46, 1.90)
Gliricidia sepium	2.77 (2.60, 2.96)
Robinia pseudoacacia	2.39 (2.23, 2.53)

Table S3 | Conversion factors for paired ARACAS and $^{15}N_2$ incubations on severed nodules. The sample size (n) for each species is the sample size after outliers were removed. This table can also be found in Bytnerowicz *et al*. (2022) *Nature Plants*, Supplementary Table 13.

	Conversion Factor (mol C_2H_4 mol N_2^{-1})	
Plant Species	Mean	SE
Morella cerifera (n=17)	4.98	0.39
Alnus rubra (n=14)	3.15	0.40
Gliricidia sepium (n=13)	3.84	0.47
Robinia pseudoacacia (n=15)	4.27	0.33

Table S4 | Parameter values for best-fit models of Eq. S1 for apparent photosynthesis as a function of CO_2. The table shows fitted values [and 95% CI] of *a*, *b* and *c* fit to individual plants. The uncertainty in *b* and *c* is used to calculated uncertainty in apparent photosynthesis rates of experimental plants (Fig. S6–S9) via parametric bootstrapping.

Plant Species	Growing Temperature	*a* (95% CI) [μCO_2 m^{-2} s^{-1}]	*b* (95% CI) [ppm CO_2]	c (95% CI) [ppm CO_2]	*a* to normalize to 1 at 400 ppm CO_2 [unitless]
G. sepium	21/15 °C	28.0<br>(8.9, 47.4)	2440<br>(2437, 2442)	−94.0<br>(−161.0, −26.8)	5.29
G. sepium	31/25 °C	59.6<br>(31.9, 87.6)	1573<br>(395, 2750)	88.6<br>(44.8, 133)	5.97
R. pseudoacacia	21/15 °C	30.6<br>(26.1, 35.2)	887<br>(642, 1130)	85.4<br>(63.2, 108)	3.82
R. pseudoacacia	31/25 °C	13.5<br>(13.1, 13.9)	63.8<br>(50.9, 76.7)	174<br>(161, 187)	1.28
M. cerifera	21/15 °C	10.7<br>(9.9, 11.4)	595<br>(496, 694)	56.3<br>(39.2, 73.1)	2.73
M. cerifera	31/25 °C	36.5<br>(33.9, 39.1)	595<br>(496, 694)	56.3<br>(39.2, 73.1)	2.73
A. rubra	21/15 °C	28.5<br>(26.2, 30.9)	291<br>(215, 366)	120<br>(97.2, 143)	2.04
A. rubra	31/25 °C	38.7<br>(35.5, 42.0)	291<br>(215, 366)	120<br>(97.2, 143)	2.04

Table S5 | ΔAIC$_c$ values for down-regulation of N fixation. The lagged sigmoid function (equation 5) has two parameters (r and t_L) that were fit at different levels of organization. The output of the best model (in bold) for r was "biome x temperature", where r differs for temperate plants grown at the cold temperature, temperate plants grown at the warm temperature, tropical plants grown at the cold temperature, and tropical plants grown at the warm temperature. This "biome x temperature" model for r was used to find the best model for t_L: the "null" model; where the same t_L applies to all plants. Since the ΔAIC$_c$ was less than 2 between competing models that varied in complexity for both r and t_L, we used likelihood ratio tests to see if the use of more complex models (lower AIC$_c$) was supported. For r, the "biome x temperature" model was significantly better than the "biome" model (p = 0.01). For t_L, the "symbiosis" model was not significantly better than the "null" model (p = 0.13). Thus, we used the "null" model for t_L, where t_L did not vary by symbiosis, temperature, or biome of origin. Parameter values for down-regulation are displayed in Table S7.

Parameter	Model	ΔAIC$_c$
r (n = 272)	**biome x temperature**	**0**
	biome	1.3
	temperature	1.6
	symbiosis	1.8
	null	2.3
	symbiosis x temperature	2.7
	species	2.7
	species x temperature	13.5
t_L (n = 32)	symbiosis	0
	**null**	**0.1**
	temperature	0.9
	biome	1.9
	symbiosis x temperature	2.5
	species	2.7
	biome x temperature	5.7
	species x temperature	12.1

Table S6 | ΔAIC$_c$ values for up-regulation of N fixation. The lagged sigmoid function (equation 5) has two parameters (r and t_L) that were fit at different levels of organization. The output of the best model (in bold) for r ("symbiosis," where the r value for rhizobial trees differs from the r value for actinorhizal trees) was used to find the best model for t_L ("symbiosis"). Since the ΔAIC$_c$ was less than 2 between competing models that varied in complexity for r, we used a likelihood ratio test to see if the use of the more complex model (lower AIC$_c$) was supported. The "symbiosis x temperature" model was only marginally better than the "symbiosis" model ($p = 0.051$). The marginally significant difference suggests that there is some support for an interaction between symbiosis and temperature, however it is weak. Thus, we used the conservative approach of using the simpler "symbiosis" model for r. Parameter values for up-regulation are displayed in Table S8.

Parameter	Model	ΔAIC$_c$
r (n = 515)	symbiosis x temperature	0
	**symbiosis**	**0.6**
	species	2.6
	species x temperature	4.7
	biome x temperature	21.7
	temperature	34.8
	biome	36.9
	null	40.4
t_L (n = 33)	**symbiosis**	**0**
	symbiosis x temperature	3.1
	species	4.3
	species x temperature	6.1
	biome x temperature	11.5
	biome	14.4
	null	15.0
	temperature	17.4

Table S7 | Parameter values for best-fit model of Eq. 5 for down-regulation of SNF.

Biome	Growing temperature	r (95% CI) [day^{-1}]	t_L (95% CI) [days]
Temperate	21/15 °C	−0.120 (−0.155, −0.084)	8.0 (3.9, 12.0)
Temperate	31/25 °C	−0.142 (−0.178, −0.108)	8.0 (3.9, 12.0)
Tropical	21/15 °C	−0.078 (−0.096, −0.061)	8.0 (3.9, 12.0)
Tropical	31/25 °C	−0.118 (−0.136, −0.099)	8.0 (3.9, 12.0)

Table S8 | Parameter values for best-fit model of Eq. 5 for up-regulation of SNF.

Symbiosis	r (95% CI) [day^{-1}]	t_L (95% CI) [days]
Actinorhizal	0.279 (0.232, 0.336)	128 (113, 143)
Rhizobial	0.104 (0.086, 0.125)	79.7 (66.2, 93.4)

Fig. S1 | Rinse test demonstrating that the growing medium (crushed granite called Gran-I-Grit Starter; North Carolina Granite Corporation, NC) does not bind NH_4^+ (teal) and NO_3^- (orange) tightly. Fertilization involved adding 30 µg N g^{-1} dry growing medium in the form of ammonium nitrate. Rinsing involved pouring 3 L of water through a 1 L pot (identical to the pots used to grow plants) filled with the growing medium. Samples were extracted in 2 M KCl and analyzed for nitrate and ammonium on a Smartchem 170 discrete analyzer (Westco Scientific Instruments, Milford MA). Each treatment (i.e. Control, Fertilized, 3 L Rinse) was replicated three times. Each measurement with the discrete analyzer was replicated twice.

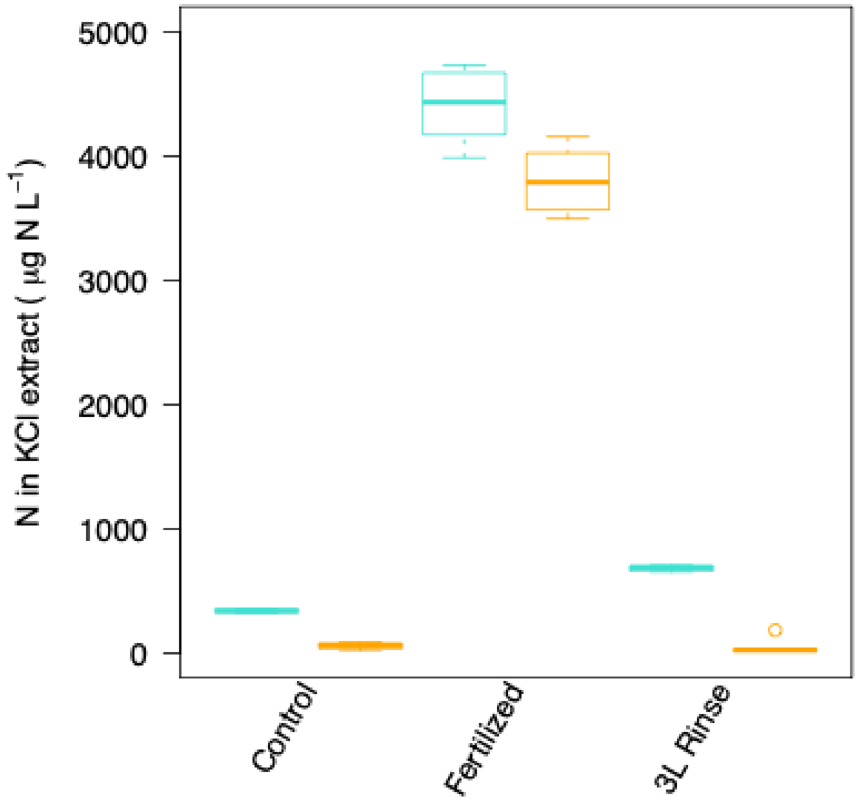

Fig. S2 | Down-regulation of N fixation as a function of time since switching to high N supply. Details as in Fig. 1, except that the absolute rates of N fixation per whole-symbiosis respiration are plotted rather than normalizing to a maximum of 1. N fixation is expressed relative to whole-symbiosis respiration to account for differences in plant size.

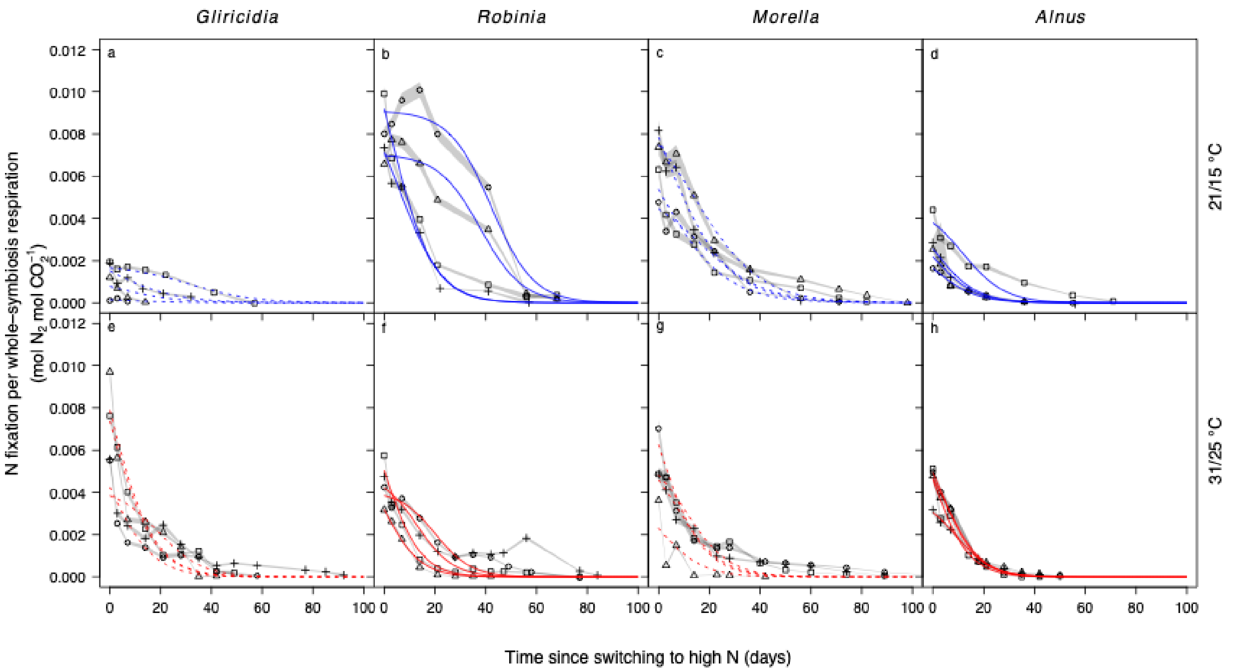

Fig. S3 | Up-regulation of N fixation as a function of time since switching to low N supply. Details as in Fig. 2, except that absolute rates of N fixation per whole-symbiosis respiration are plotted rather than rates normalized to 1.

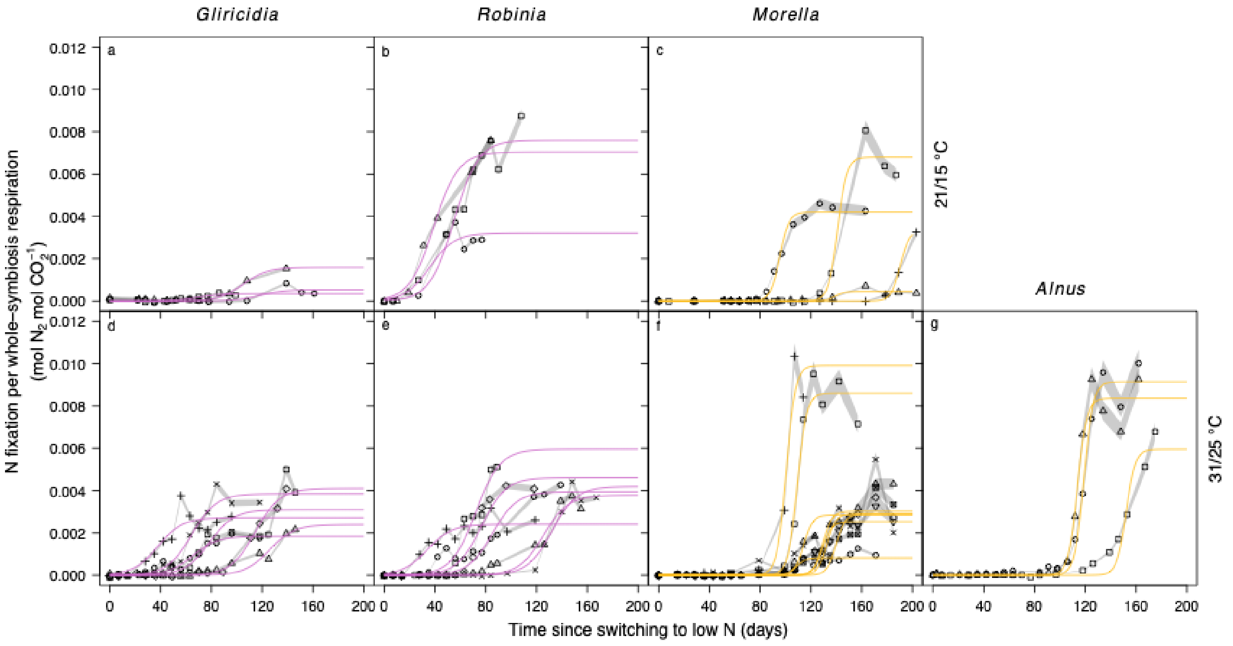

Fig. S4 | Down-regulation of N fixation as a function of time since switching to high N supply. Details as in Fig. S2, except that N fixation is not divided by whole-symbiosis respiration.

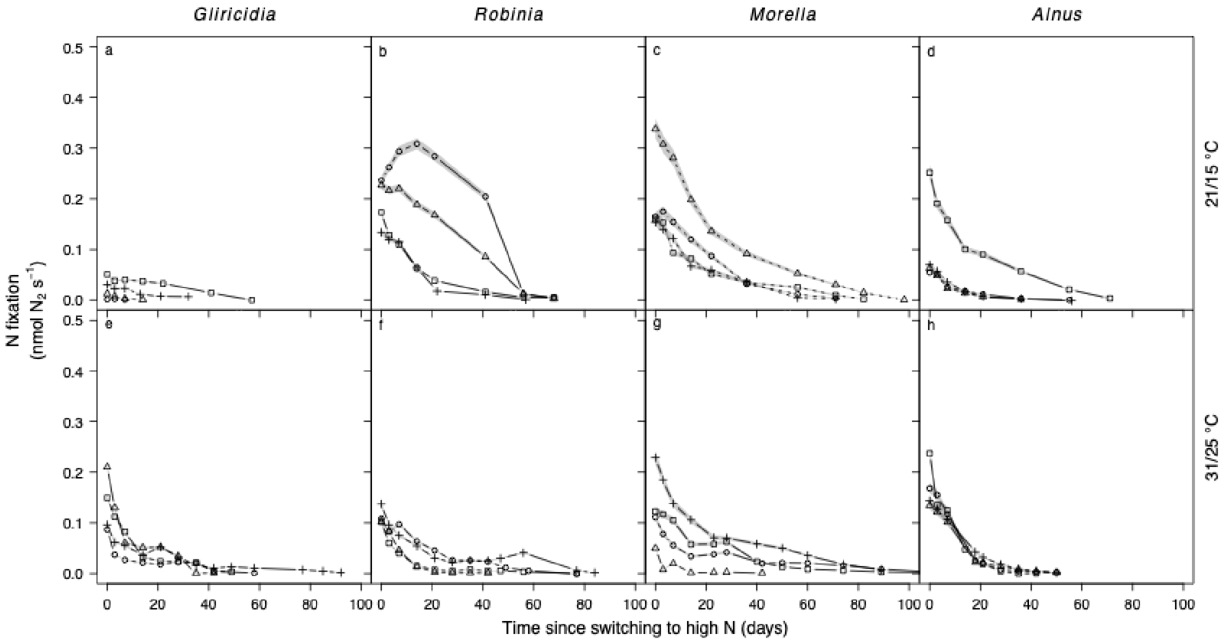

Fig. S5 | Up-regulation of N fixation as a function of time since switching to low N supply. Details as in Fig. S3, except that N fixation is not divided by whole-symbiosis respiration.

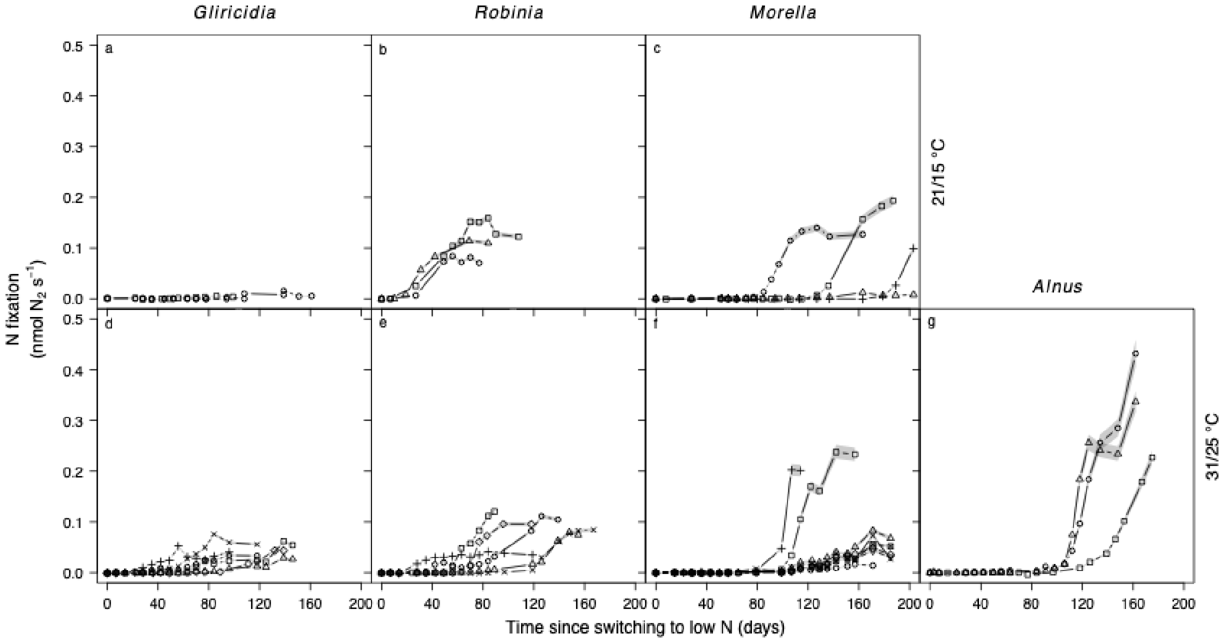

Fig. S6 | Apparent whole-plant photosynthesis following a switch of plants to high N supply. Data are normalized to 1 at day = 0. Different points within each pane represent individual plants and the gray shading represents 95% CI values, as in Figs. 1, 2, S2–S5. The red horizontal line shows the relative photosynthesis rate at day = 0.

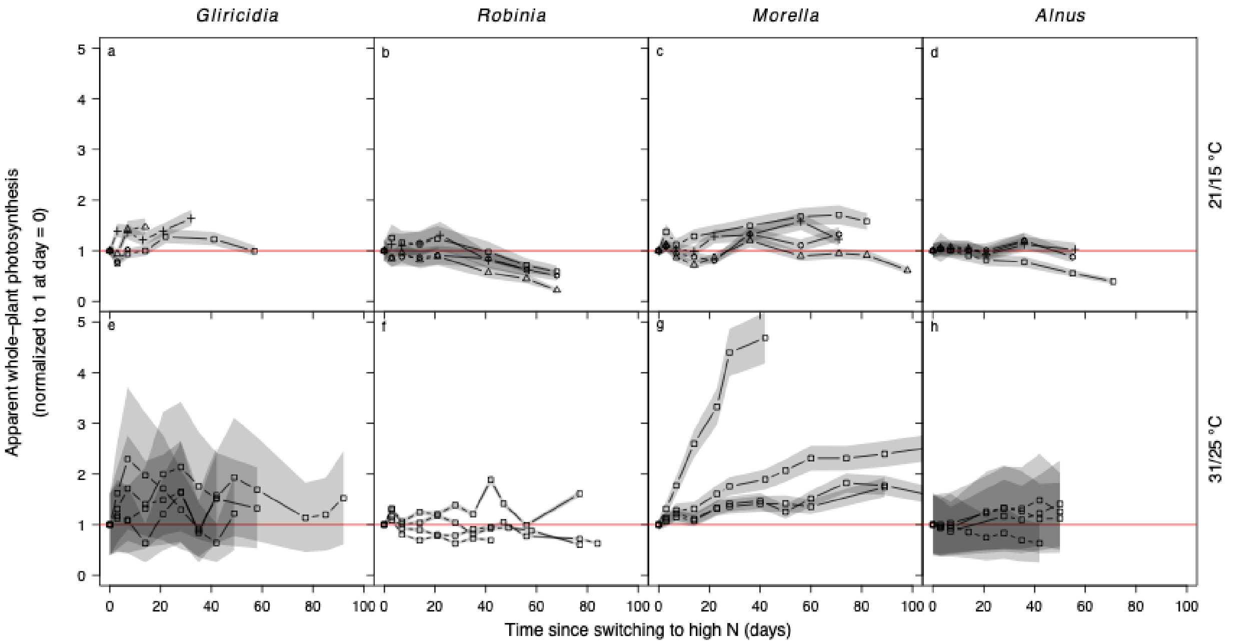

Fig. S7 | Apparent whole-plant photosynthesis following a switch of plants to high N supply. Details as in Fig. S6, except absolute rates are plotted rather than rates normalized to 1.

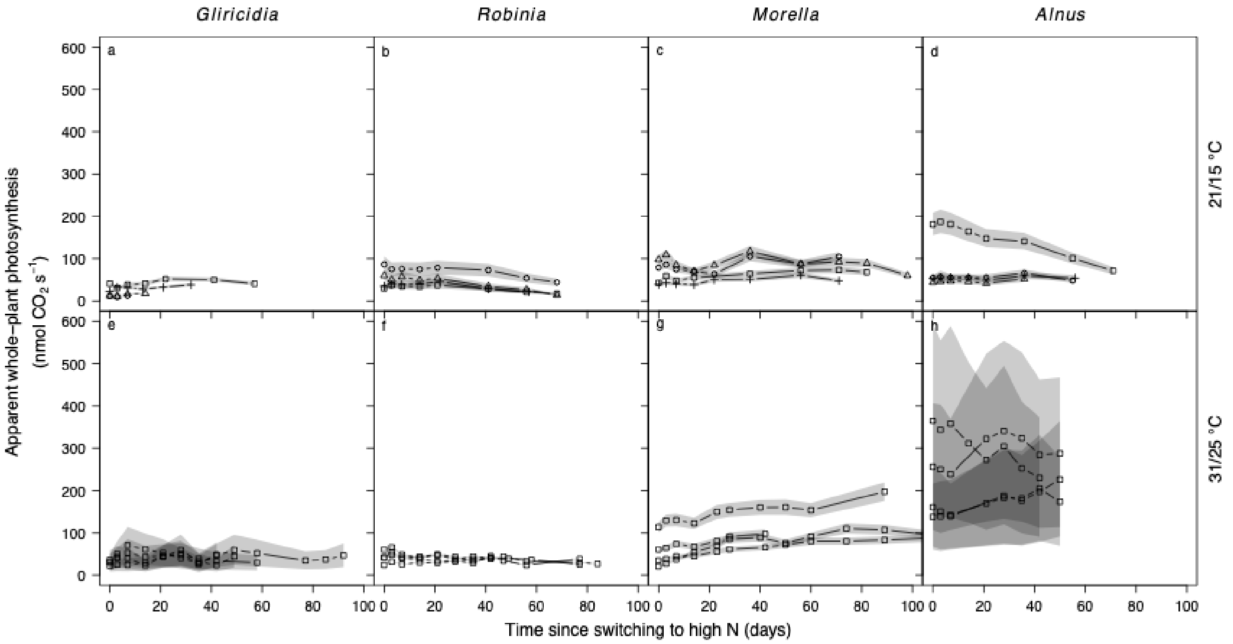

Fig. S8 | Apparent whole-plant photosynthesis following a switch of plants to low N supply. Details as in Fig. S6.

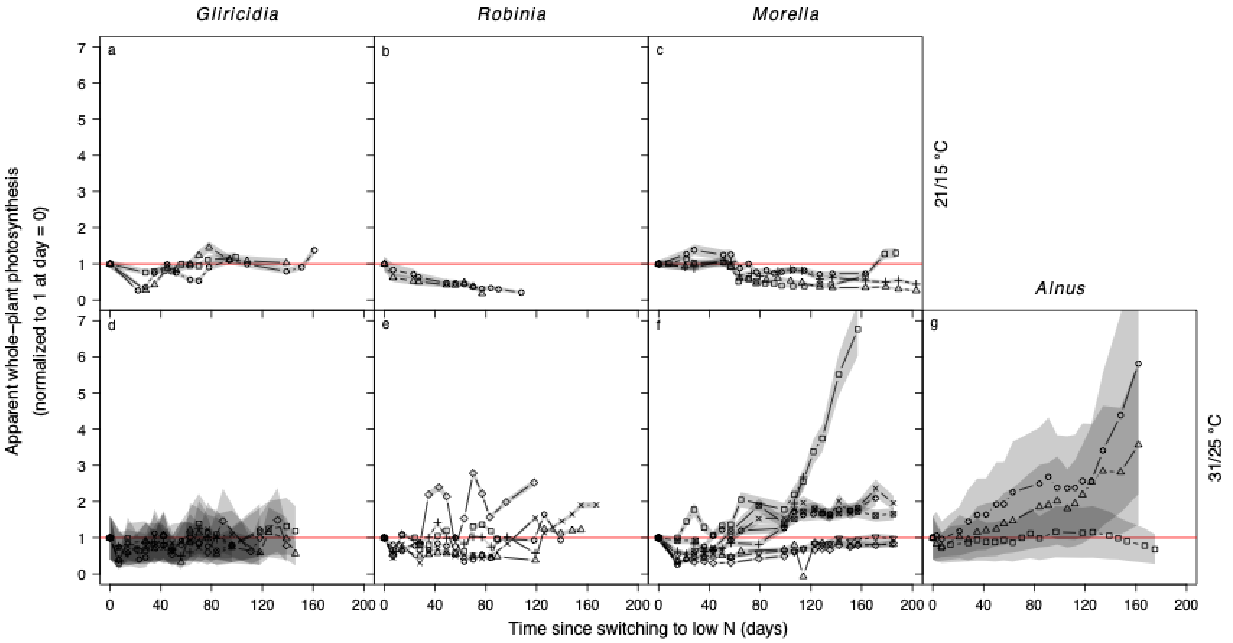

Fig. S9 | Apparent whole-plant photosynthesis following a switch of plants to low N supply. Details as in Fig. S8, except absolute rates are plotted rather than rates normalized to 1.

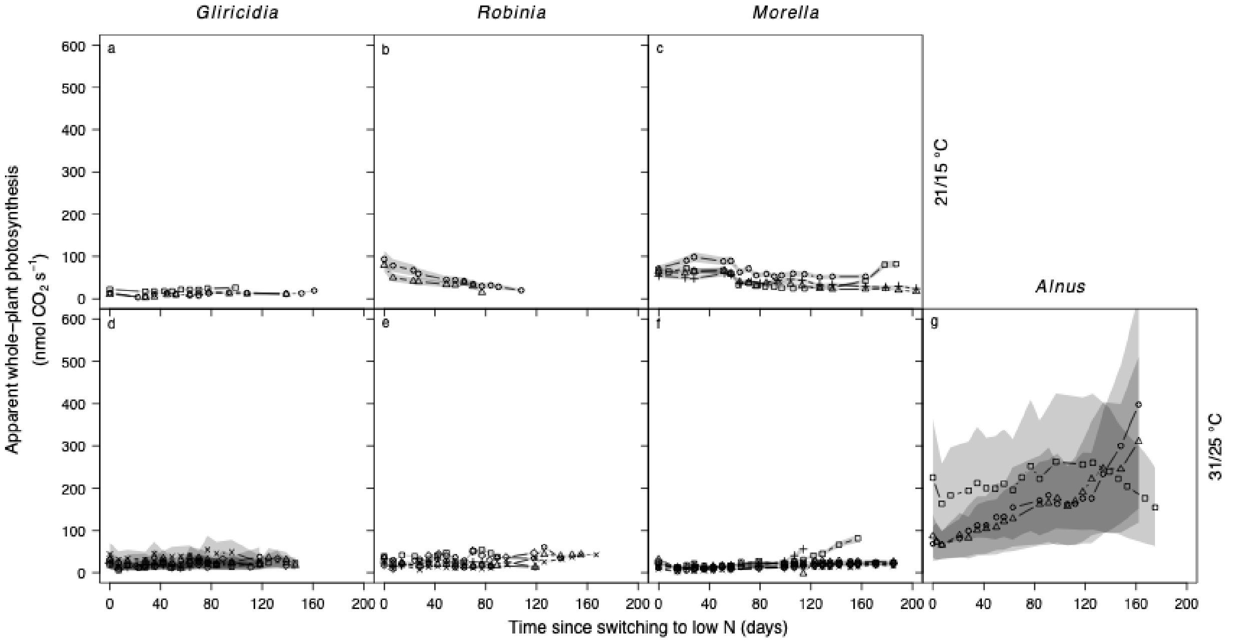

Fig. S10 | Whole-symbiosis respiration following a switch of plants to high N supply. Details as in Fig. S6.

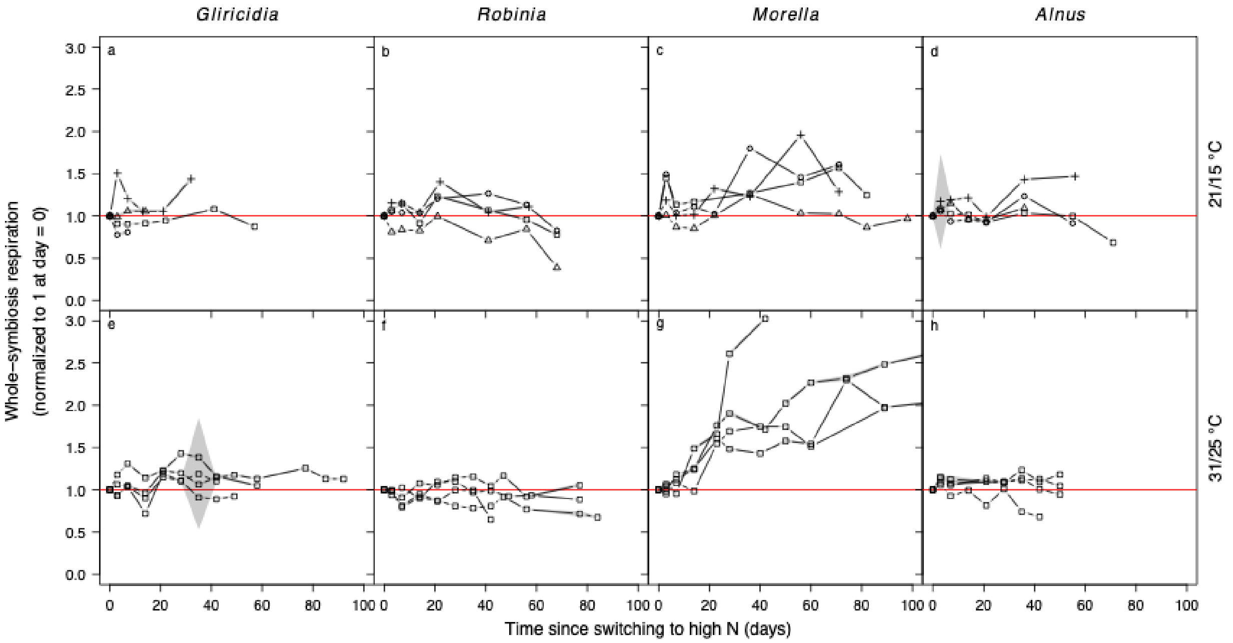

Fig. S11 | Whole-symbiosis respiration following a switch of plants to high N supply. Details as in Fig. S10, except absolute rates are plotted rather than rates normalized to 1.

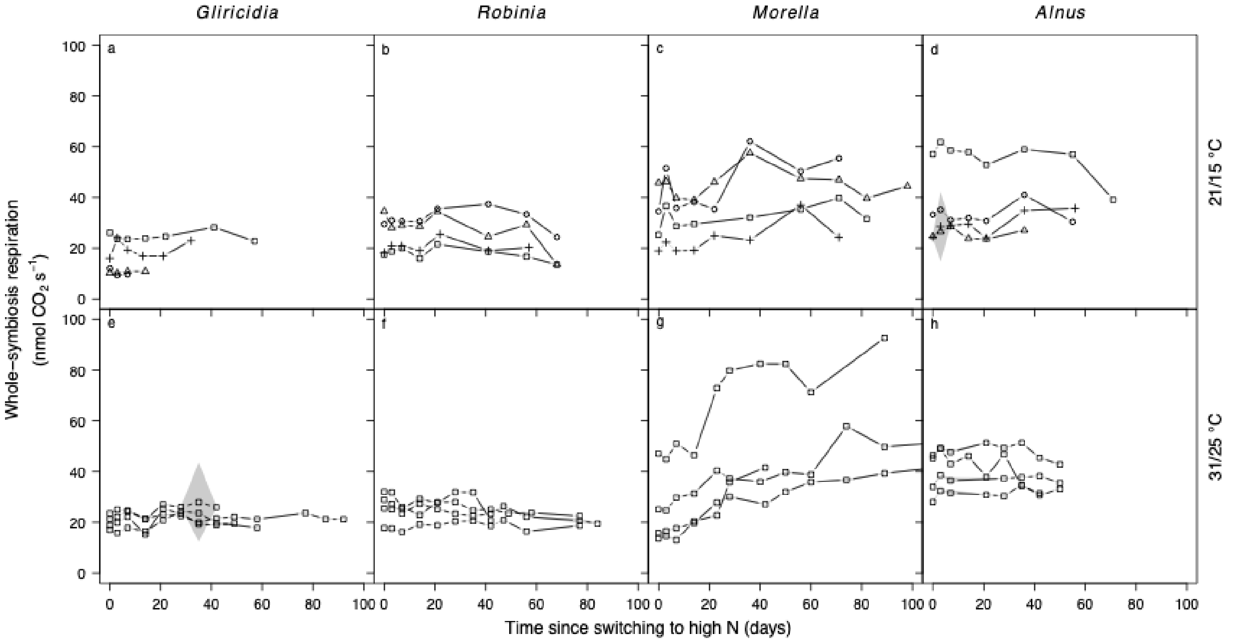

Fig. S12 | Whole-symbiosis respiration following a switch of plants to low N supply. Details as in Fig. S6.

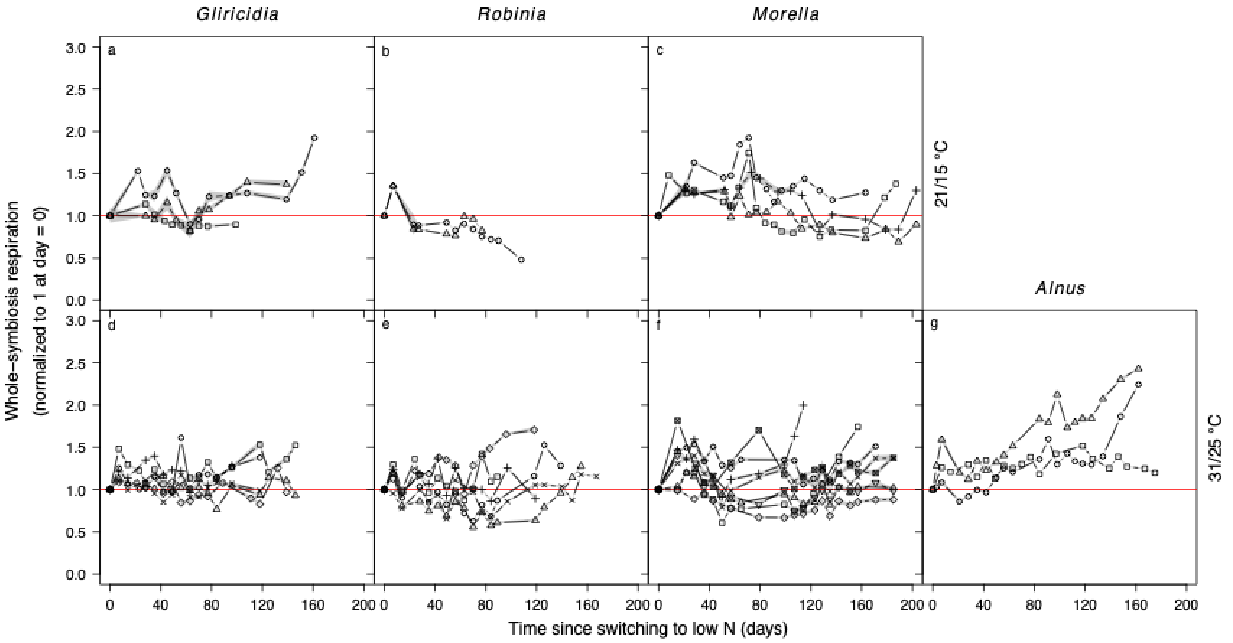

Fig. S13 | Whole-symbiosis respiration following a switch of plants to low N supply. Details as in Fig. S12, except absolute rates are plotted rather than rates normalized to 1.

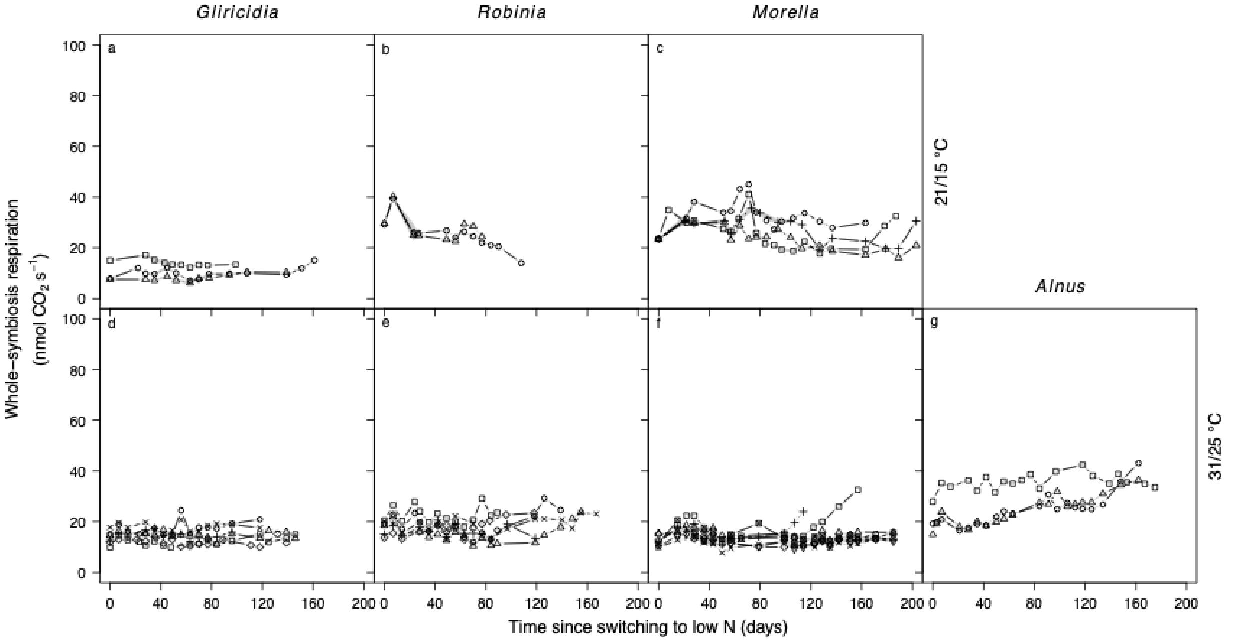

Fig. S14 | The t_L parameter from Eq. 5 as a function of the whole-symbiosis respiration rate in plants that were down-regulating SNF. Dashed curves represent the 95% confidence interval.

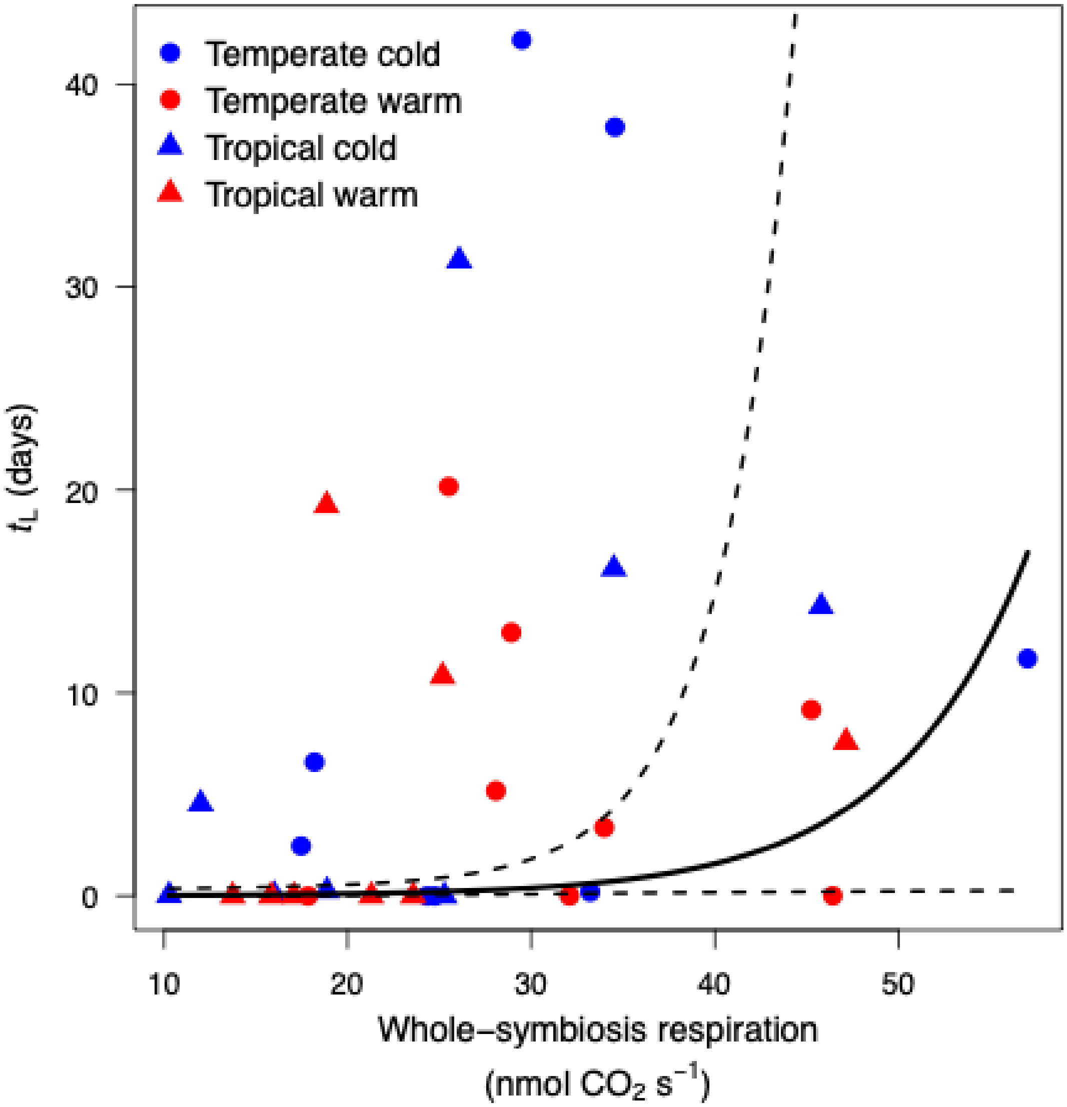

References

Bolker BM, R Core Team. 2014. bbmle: Tools for General Maximum Likelihood Estimation.

Bytnerowicz TA, Akana PR, Griffin KL, Menge DNL. **2022**. Temperature sensitivity of woody nitrogen fixation across species and growing temperatures. *Nature Plants* **8**: 209–216.

Bytnerowicz TA, Min E, Griffin KL, Menge DNL. **2019**. Repeatable, continuous and real-time estimates of coupled nitrogenase activity and carbon exchange at the whole-plant scale. *Methods in Ecology and Evolution* **10**: 960–970.
